# Physics informed neural network surrogate modeling and global sensitivity analysis of magnetized nanofluid flow over a porous cylinder for solar thermal energy applications

**DOI:** 10.1186/s11671-026-05023-5

**Published:** 2026-10-28

**Authors:** Mohib Hussain, Abdullah Alhushaybari

**Affiliations:** 1 State Key Laboratory of Intelligent Construction and Healthy Operation and Maintenance of Deep Underground Engineering, College of Civil and Transportation Engineering, Shenzhen University, Shenzhen, China; 2 Department of Mathematics, College of Turabah, Taif University, Taif, 21944 Saudi Arabia

**Keywords:** Physics-informed neural networks, Predictive surrogate modeling, Global sensitivity analysis, Porous media, Thermal prediction, Magnetized nanofluid

## Abstract

The accurate prediction of mass and heat transfer dynamics in the movement of magnetized nanofluids over porous cylindrical structures is crucial for the design and improvement of innovative solar thermal energy systems. However, traditional numerical methods need comprehensive high-fidelity simulations over several parameter domains, limiting their use in real-time evaluation, optimization, and decision-making. Physics-informed neural networks (PINNs) are becoming dependable alternatives to traditional computational fluid dynamics (CFD) methods for modeling nonlinear fluid dynamics in aerospace and medical engineering. In this study, we introduced a hybrid framework that leverage PINNs, and an adapted finite difference method to improve the computational analysis of nonlinear magnetized ternary nanofluid flow produced by a permeable stretching cylinder. The investigated working fluid is composed of multi-walled carbon nanotubes, A7075, and graphene oxide nanoparticles suspended in a blend of ethylene glycol and water.The framework of the PINNs, which includes a defined number of hidden layers, a training algorithm, a learning rate, and an epoch count, has been determined through systematic hyper-parameter optimization. We additionally integrated the dynamic influences of magneto hydrodynamic (MHD) phenomena, activation energy, and thermal radiation to enhance heat transfer and regulate fluid flow phenomena. The established set of mathematical equations are addressed using a modified finite difference method and PINNs to accurately predict fluid flow characteristics. The global sensitivity analysis (GSA) was performed to demonstrate the sensitivity and impact of governing factors on fluid dynamics. Thermal radiation and the Biot number strongly influence temperature variance, while the concentration of nanoparticles acts as a governing factor that exerts significant impacts on velocity and heat transmission. The persistently low PINNs loss metrics, remaining below ${10}^{-11}$, indicate exceptional computational precision and validate the great fidelity of the suggested architecture in accurately representing the controlling thermodynamics of the system. Moreover, the DNN-enforced PINNs approach efficiently corresponds to solutions for an intricate thermal-fluid framework with coefficient of determination $({R}^{2}\mathrm{) > 0.99}$. The established PINN surrogate delivers rapid predictions post-training, significantly alleviating the computing cost linked to recurrent high-fidelity numerical simulations, thus presenting an effective option for parametric analysis and global sensitivity evaluation.

## Introduction

Space exploration projects provide problems to worldwide space organizations due to escalating power consumption and radiating heat needs. Strategies for enhancing heat transfer seek to optimize power efficiency while minimizing size and weight. Heat transmission in space is essential in domains such as electronic cooling, aviation engine cooling, protection from heat, and control over the environment equipment [1]. Nonetheless, the processes of heat convection as well as radiation in space remain un-examined. Advancement of heat and environmental management theories is essential to enhance the efficiency of aircraft and aerospace engineering. The process of heat transfer through the movement of fluids, known as convection [2], presents a practical and efficient approach to thermal oversight in aerospace environments, minimizing the necessity for intricate and costly cooling mechanisms. The conventional liquids, such as water, and engine oil, are typically employed as heat convection fluids in a variety of heat transfer applications [3]. Despite the application of different methods to enhance heat transfer, their implementation remains constrained by restricted thermal conductivity, which hinder performance improvement and lead to inadequate thermal exchangers. However, in contrast to conventional liquids, nanofluids exhibit significantly enhanced thermal conductivity [4, 5]. The term "nanofluid" was first coined by Choi [6]. Nanofluid represents a distinct category of fluid, meticulously crafted through the dispersion of nanoparticles, which are on the manometer scale, within a base fluid [7]. When combined with the base liquid, nanofluids demonstrate distinct and improved characteristics in comparison to the base liquid by itself [8]. In recent years, researchers, introduced new kinds of nanofluids known as "hybrid nanofluid" and "tri-hybrid (ternary) nanofluid" [9, 10]. Hybrid nanofluids consist of two types of nanoparticles, whereas ternary nanofluids contain three distinct nanoparticles, all dispersed within the base fluid at the same time [11]. Mumtaz et al. [12] conducted an investigation into the water-based ternary nanofluid, focusing on its behavior in curved topology while considering cross-diffusion. The findings indicate that heat conductivity rises from $\mathrm{6 \% }$ to $\mathrm{26 \% }$ as the volume percentage of nanoparticles is increased from $\mathrm{1 \% }$ to $\mathrm{4 \% }$. Hussain et al. [13] examine the improvement in mass and heat transfer utilizing a ternary nanofluid created using kerosene oil, with an emphasis on flow through a porous media. Mahboobtosi et al. [14] studied the movement of kerosene oil using ternary mixed nanoparticles on a stretching surface. The findings demonstrate that skin friction increases as the volume percentage of nanoparticles increases for a given value of the magnetic field. For the purpose of cooling rocket engines, Sayed et al. [15] investigated the heat transmission study of ternary nanofluid flow under second slip circumstances. The ternary nanofluids have remarkable thermal performance, yet, how they act during shear stress and non-Newtonian flow circumstances is a crucial area of investigation. This encourages consideration of the Williamson fluid framework, which offers a more accurate representation of complex nanofluid dynamics in actual applications by including shear-thinning impacts and viscoelastic properties.

The study of boundary layer flow, mass, and thermal transmission of non-Newtonian liquids across a stretching cylinder holds significant importance for professionals in various fields. This topic is relevant in aerospace engineering due to its numerous applications, rotating turbine blades, and curvature of nose cones or leading edges. The Williamson fluid model exhibits different notable characteristics among various fluids model [16]. It is applicable to liquids with a low viscosity along with those having high viscosity. Hayat et al. [17] studied the effects of thermal radiation, and magnetic field on the boundary layer flow of Williamson fluid along a stretched cylinder. Waqas et al. [18] documented the mixed convection movement of the Williamson liquid in an in-compressible, steady way across a flat, stretched surface. Song et al. [19] investigated the unsteady movement of nanofluid induced by a permeable stretched cylinder with variable thermal characteristics. Zaman et al. [20] examined transportation of heat in the movement of Williamson fluid model-based nanofluid, considering the impact of magnetohydrodynamics (MHD) and radiation along a thin cylinder. Fareed et al. [21] investigated the Darcy−Forchheimer movement of Williamson ternary hybrid nanofluid across a stretched cylinder. They employed the aluminium oxide, titanium oxide, and silicon dioxide nanoparticles in base fluid ethylene glycol. Analyzing the actions and conduct of non-Newtonian fluids, shown by the Williamson model, often involves addressing highly nonlinear PDEs that provide significant computational challenges. Despite their efficiency, traditional numerical methods may struggle with complex variable actual time optimization scenarios. ML methodologies particularly PINNs, GNN, and Artificial Neural Networks (ANNs) have shown efficacy as robust instruments for simulating complex fluid dynamics (CFD) to circumvent these limitations. These approaches provide a promising alternative for the rapid and precise modeling of nanofluid dynamics via data-driven learning, while adhering to basic physical constraints.

In recent years, ML has achieved notable successes, largely due to breakthroughs in graphics processing units and the strong development of programming platforms. It is used in several domains, including physics, linguistics, biology, as well as in situations such as image recognition, natural language processing, and autonomous driving [22, 23]. Inspired by these breakthroughs, applied mathematicians have swiftly developed several creative frameworks using machine learning, aiming to revolutionize the traditional model of quantitative engineering and science [24, 25]. The innovative frameworks may include data-driven, and physics-informed-regularized approaches, and involving the optimization of discrete loss, among others [26, 27]. Specifically, PINNs have catalyzed extensive research initiatives [28]. The fundamental concept of PINNs is to integrate the residuals of PDEs into the neural network loss function and use its robust non-linear fitting capabilities to approximate solutions [29]. Scholars have expanded the use of PINNs to address diverse categories of differential equations [30, 31]. Aslam et al. [32] examines the effects of different nanoparticle and heat transfer characteristics on magnetohydrodynamics (MHD) nano-fluid flow. A mathematical model utilizing PINNs and the Levenberg−Marquardt method was employed to address the problem. Chandra et al. [33] employed PINNs to investigate bio-convection nanofluid flow across an inclined stretched surface. A structured PINNs architecture was developed to enforce the starting and boundary requirements. Jawad et al. [34] employed PINNs to analyze the rotating electromagnetic hydrodynamic flow of Jeffrey hybrid nanofluid, incorporating activation energy.

The literature review emphasizes the significance of precise modeling of heat transport and fluid dynamics issues in both engineering and science. However, only computational and analytical methods are accessible for resolving intricate coupled geometries. Traditional CFD techniques yield reliable results just when fields exhibit smooth variations across the grid. This motivate us to investigate ML methodologies to establish an accurate, robust, and reliable computational technique for addressing CFD challenges. A novel PINNs and DNN approach combined with modified finite difference approach, is proposed for the analysis of ternary nanofluid flow across a stretchable cylinder. The DNN-enforced PINNs offer a robust framework for addressing the non-linear ODEs governing Williamson model ternary nanofluids, and their application to intricate multi-physics systems, particularly in contexts involving non-Newtonian rheology, thermal transport, and nanoparticle interactions. Our methodology enhances the precision and efficacy of simulating ternary nanofluid dynamics while facilitating real-time optimization for industrial cooling systems through numerical approximation utilizing PINNs. Furthermore, a global sensitivity inspection (GSA) was conducted to elucidate the impact of regulating parameters on the coupled system of mathematical expressions, indicating that the curvature parameter in conjunction with nanoparticle density, the Biot number alongside heat radiation, and the curvature parameter with nanoparticle concentration are the most influential factors for the controlling equations of velocity, temperature, and concentration. Conclusively presented study enhances the computational modeling of non-Newtonian nanofluids by correlating theory-driven and high-dimensional parameter spaces with physical constraints. The computational outcomes derived from the current study can be beneficial across various engineering domains, as the engine oil/water nanofluid mixture functions as an efficient coolant in aviation and high-tech electronic cooling mechanisms, offering a smart blend of antifreeze stability and enhanced thermal conductivity for crucial heat dissipation.

## Mathematical formulation

**Fig. 1:**
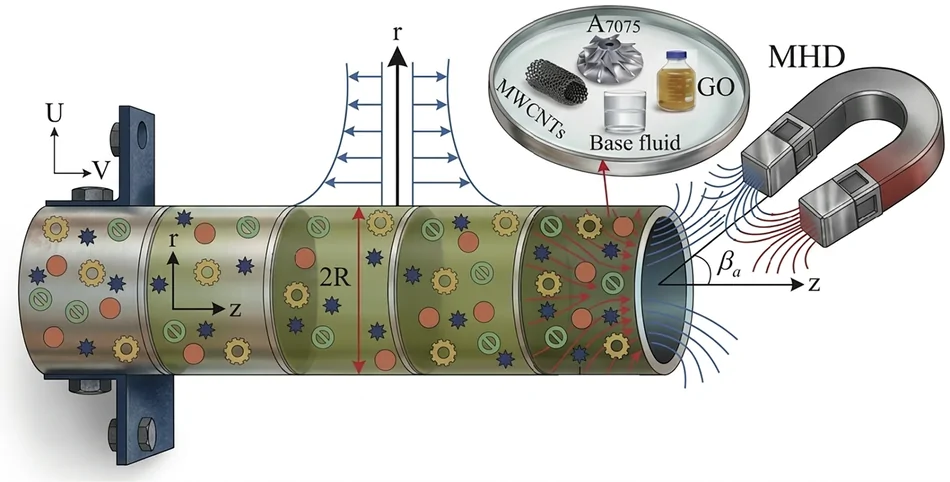
Physical description

Let us consider an in-compressible Williamson model ternary nanofluid flow influenced by a stretching cylinder, as depicted in Fig. 1. The cylinder undergoes axial stretching according to the prescribed wall velocity $\frac{{U}_{0}z}{l}$, where l denotes the characteristic length of the cylinder. We make following assumptions to formulate the governing equations:

- The fluid flow is assumed to be steady and in-compressible.
- The applied magnetic field $({B}_{0})$ in inclined at an angle ${\beta }_{a}$ with respect to the axial z-direction.
- The Williamson fluid model is used to formulate the ternary nanofluid flow phenomena.
- The impacts of Darcy-Forchheimer, and thermal radiation are also considered.
- $EG/W\mathrm{(60/40)}$ is employed as base fluid whereas, multi-walled carbon nanotubes, A7075, and graphene oxide (GO) are the dispersed nanoparticles.
- Convective boundary constraints are assumed to enhance the realism of physical processes.
- The velocity field is represented by U(r,z) and V(r,z), where U is the radial velocity component in the r-direction and V is the axial velocity component in the z-direction.
- Owing to the low magnetic Reynolds number, induced magnetic field negligible relative to applied magnetic force.

Considering all the aforementioned suppositions the governing equations for the scenario at consideration are [35]:

1 $$\frac{\partial (rU)}{\partial r}+\frac{\partial (rV)}{\partial z}\mathrm{=0,}$$

2 $$\begin{array}{ll}{\rho }_{tnf}\left[U\frac{\partial V}{\partial r}+V\frac{\partial V}{\partial z}\right]= & {\mu }_{tnf}\left[\frac{{\partial }^{2}V}{\partial {r}^{2}}+\frac{1}{r}\frac{\partial V}{\partial r}+\sqrt{2}\varGamma \frac{\partial V}{\partial r}\frac{{\partial }^{2}V}{\partial {r}^{2}}+\frac{\varGamma }{\sqrt{2}r}{(\frac{\partial V}{\partial r})}^{2}\right]\\ & -{\sigma }_{bf}{B}_{0}^{2}V{\sin }^{2}({\beta }_{a})-\frac{{\mu }_{tnf}V}{{k}^{\ast }}-\frac{{\rho }_{tnf}{F}_{c}}{\sqrt{{k}^{\ast }}}{V}^{2},\end{array}$$

3 $$U\frac{\partial H}{\partial r}+V\frac{\partial H}{\partial z}=\frac{{\alpha }_{tnf}}{r}\frac{\partial }{\partial r}\left(r\frac{\partial H}{\partial r}\right)-\frac{1}{{(\rho {C}_{p})}_{tnf}}\frac{1}{r}\frac{\partial }{\partial r}(r{q}_{r}),$$

4 $$U\frac{\partial C}{\partial r}+V\frac{\partial C}{\partial z}={D}_{bf}\frac{1}{r}\frac{\partial }{\partial r}\left(r\frac{\partial C}{\partial r}\right)-{K}_{r}^{2}{\left(\frac{H}{{H}_{\infty }}\right)}^{n}\exp \left(-\frac{E}{{k}_{B}H}\right)\left(C-{C}_{\infty }\right).$$

The corresponding boundary constraints are [35, 36]:

5 $$U\mathrm{=0,}\,V=\frac{{U}_{0}z}{l},\,{k}_{tnf}\frac{\partial H}{\partial r}=-{h}_{w}({H}_{w}-H),\,C={C}_{w}\,{\rm{at}}\,r=R$$

6 $${\rm{when}}\:r\to \infty ,\,U\to \mathrm{0,}\,C\to {C}_{\infty },\,H\to {H}_{\infty }\mathrm{}.$$

Here, H and C represent temperature and concentration, respectively. Additionally, ${K}_{r}^{2},E,{H}_{\infty }$, and ${C}_{\infty }$ indicate reaction rate constant, activation energy constant, ambient temperature, and concentration. The following similarity scaling is employed to shift governing PDEs into ODEs [37]:

7 $$\begin{array}{lll}\eta &=&\left(\frac{{r}^{2}-{R}^{2}}{2R}\right)\sqrt{\frac{{U}_{0}}{{\nu }_{bf}l}}, \quad U=-f(\eta )(\frac{R}{r})\sqrt{\frac{{U}_{0}{\nu }_{bf}}{l}},\,\theta (\eta )=\frac{{H}_{\infty }-H}{{H}_{\infty }-{H}_{w}}\\ \phi (\eta )&=&\frac{C-{C}_{\infty }}{{C}_{w}-{C}_{\infty }},\quad V=f{\prime} (\eta )\frac{{U}_{0}z}{l}\mathrm{}.\end{array}$$

By using the similarity shifts, Eq. 1 is proved identically and Eqs. 2 - 4 are reformulated as:

8 $$\begin{array}{lll}\mathrm{(2}{\gamma }_{c}\eta +\mathrm{1)}f{\prime\prime} {\prime} +2{\gamma }_{c}f{\prime\prime} +{\alpha }_{w}{\mathrm{(2}{\gamma }_{c}\eta +\mathrm{1)}}^{1.5}f{\prime\prime} {\prime} f{\prime\prime} +1.5{\gamma }_{c}{\alpha }_{w}\sqrt{\mathrm{(2}{\gamma }_{c}\eta +\mathrm{1)}}{(f{\prime\prime} )}^{2}\\ -{P}_{2}M{\sin }^{2}({\beta }_{a})f{\prime} -{K}_{p}f{\prime} +{P}_{1}(f{\prime} {\prime} f-{f{\prime} }^{2}-Fr{f{\prime} }^{2}\mathrm{)=0,}\end{array}$$

9 $$\left(\frac{4{R}_{d}}{3}+{P}_{4}\right)\mathrm{(2}{\gamma }_{c}\eta +\mathrm{1)}\theta {\prime\prime} +2\left(\frac{4{R}_{d}}{3}+{P}_{4}\right){\gamma }_{c}\theta {\prime} +{P}_{3}{P}_{r}\theta {\prime} f\mathrm{=0,}$$

10 $$\mathrm{(2}{\gamma }_{c}\eta +\mathrm{1)}\phi {\prime\prime} +2{\gamma }_{c}\phi {\prime} +Sc\left(f\phi {\prime} -{C}_{r}{(1+\tilde{\delta }\theta )}^{n}\exp \left(-\frac{{E}_{a}}{1+\tilde{\delta }\theta }\right)\phi \right)\mathrm{=0}.$$

The boundary conditions are revised as;

11 $$f{\prime} \mathrm{=1,}\,f\mathrm{=0,}\,\theta {\prime} =-\mathrm{(1}-\theta )\frac{{B}_{i}}{{P}_{4}},\,\phi \mathrm{=1,}{\rm{when}},\eta \mathrm{=0,}$$

12 $$f{\prime} \to \mathrm{0,}\,\theta \to \mathrm{0,}\,\phi \to \mathrm{0,}\,{\rm{when}},\eta \to \infty \mathrm{}.$$

Where, $${\gamma }_{c}=\frac{1}{R}\sqrt{\frac{{\nu }_{bf}l}{{U}_{0}}}$$, $${F}_{r}=\frac{{F}_{c}{z}_{0}}{\sqrt{{k}^{\ast }}},$$$${\alpha }_{W}=\sqrt{2}\varGamma \frac{{z}_{0}{U}_{0}}{l}\sqrt{\frac{{U}_{0}}{{\nu }_{bf}l}},$$$${R}_{d}=\frac{4\sigma {H}_{\infty }^{3}l}{{k}_{r}{k}_{bf}},$$$${P}_{r}=\frac{{\nu }_{bf}}{{\alpha }_{bf}},$$$${E}_{a}=\frac{E}{{k}_{B}{H}_{\infty }},$$$${K}_{p}=\frac{{\nu }_{bf}l}{{U}_{0}{k}^{\ast }}$$ and $${S}_{c}=\frac{{\nu }_{bf}}{{D}_{bf}}$$ indicates curvature variable, Forchheimer number, Williamson fluid parameter, thermal radiation, Prandtl, activation energy, the porosity and Schmidt number, respectively. Whereas, $${P}_{1}=\frac{{\nu }_{tnf}}{{\nu }_{bf}},{P}_{2}=\frac{{\sigma }_{tnf}}{{\sigma }_{bf}},{P}_{3}=\frac{{(\rho {C}_{p})}_{tnf}}{{(\rho {C}_{p})}_{bf}}$$, $${P}_{4}=\frac{{k}_{tnf}}{{k}_{bf}}$$.

### Thermophysical characteristics of ternary nanofluid

The ternary nano-liquids comprise multi-walled carbon nanotubes (MWCNTs), A7075, and graphene oxide (GO), while mixture ethylene glycol (EG) and water (W) with percentage of $\mathrm{60:40}$ is employed as the basis liquid. The EGâ€“W blend is selected as a base liquid because of its equilibrium between thermal conductivity and flow properties, functioning efficiently as a heat-transfer agent. The chosen nanoparticles MWCNTs,A7075, and GO provide synergistic thermal characteristics. MWCNTs boost thermal conduction due to their elevated conductivity, A7075 offers a beneficial metallic phase, and GO supplies an extensive surface area for enhanced thermal properties. This amalgamation seeks to improve the thermophysical behavior of the liquid, rendering it appropriate for examining the interrelated momentum and heat-transfer properties in thermal-fluid contexts. The thermophysical characteristics of nanoparticles and base liquid are shown in Table 1. Mathematical expression to calculate thermophysical properties of ternary nanofluid is given in Table 2. The Nusselt number is a dimensionless quantity employed in fluid mechanics to assess boundary convective to conductive thermal transfer. It measures thermal transfer performance under various fluid flow situations. Higher Nusselt numbers correspond to better fluid-to-surface heat transmission. The Skin friction coefficient describes the shear stress across the fluid-solid boundary relative to fluid flow pressure fluctuations. It is important for predicting drag forces on particles moving through fluids in aerodynamics and hydrodynamics, impacting energy efficiency and productivity. Herein, the Nusselt number $({N}_{u})$, and skin-friction number $({C}_{f})$ in non-dimensional form are defined as:

13 $${C}_{f}\sqrt{{R}_{e}}=\frac{1}{{P}_{2}}\left(\frac{{\alpha }_{W}}{2}f{\prime\prime} \mathrm{(0)}+1\right)f{\prime\prime} \mathrm{(0),}$$

14 $$\frac{{N}_{u}}{\sqrt{{R}_{e}}}=-{P}_{4}\left(1+\frac{4}{3}{R}_{d}\right)\theta {\prime} \mathrm{(0)}.$$

Where ${R}_{e}$ is the Reynolds number.

**Table 1:** Thermophysical properties of base fluid and nano-particles [47]

	EG:W	MWCNTs	A7075	GO
$\rho (\frac{kg}{{m}^{3}})$	1059.68	1600	2810	1800
${C}_{p}(\frac{J}{kgK})$	3468	796	960	717
$k(\frac{W}{mK})$	0.404	3000	173	5000

**Table 2:** Thermophysical properties of ternary nanofluid [48]

Property	Mathematical Expression
Density (ρ)	$$\begin{array}{c}{\rho }_{tnf}\mathrm{=(1}-{\phi }_{MWCNTs}\mathrm{)\{(1}-{\phi }_{A7075}\mathrm{)[(1}-{\phi }_{GO}){\rho }_{bf}+{\phi }_{GO}{\rho }_{GO}]+{\phi }_{A7075}{\rho }_{A7075}\}\\ +{\phi }_{MWCNTs}{\rho }_{MWCNTs}\end{array}$$
Dynamic Viscosity (μ)	${\mu }_{tnf}=\frac{{\mu }_{bf}}{{\mathrm{(1}-{\phi }_{MWCNTs})}^{2.5}{\mathrm{(1}-{\phi }_{A7075})}^{2.5}{\mathrm{(1}-{\phi }_{GO})}^{2.5}}$
Heat Capacity ($\rho {C}_{p}$)	$$\begin{array}{c}{(\rho {C}_{p})}_{tnf}\mathrm{=(1}-{\phi }_{MWCNTs}\mathrm{)\{(1}-{\phi }_{A7075}\mathrm{)[(1}-{\phi }_{3})(\rho {C}_{p}{)}_{bf}+{\phi }_{GO}{(\rho {C}_{p})}_{GO}]\\ +{\phi }_{A7075}{(\rho {C}_{p})}_{A7075}\}+{\phi }_{MWCNTs}{(\rho {C}_{p})}_{MWCNTs}\end{array}$$
Thermal Conductivity (k)	${k}_{tnf}=\frac{{k}_{s3}+2{k}_{nf}-2{\phi }_{GO}({k}_{nf}-{k}_{GO})}{{k}_{GO}+2{k}_{nf}+{\phi }_{GO}({k}_{nf}-{k}_{GO})}\times {k}_{hnf}$
	where${k}_{hnf}=\frac{{k}_{A7075}+2{k}_{nf}-2{\phi }_{2}({k}_{nf}-{k}_{A7075})}{{k}_{A7075}+2{k}_{nf}+{\phi }_{A7075}({k}_{nf}-{k}_{A7075})}\times {k}_{nf}$
	and${k}_{nf}=\frac{{k}_{s1}+2{k}_{bf}-2{\phi }_{MWCNTs}({k}_{bf}-{k}_{MWCNTs})}{{k}_{MWCNTs}+2{k}_{f}+{\phi }_{MWCNTs}({k}_{bf}-{k}_{MWCNTs})}\times {k}_{bf}$

## Hybrid simulation approach

Non-linear problems are common across several domains, such as meteorology, economic portfolio optimization, and the comprehension of biological phenomena. High-performance computing allows numerical methods to effectively resolve these issues, empowering academics to address computationally intensive mathematical challenges that cannot be solved either numerically or analytically. The Keller-Box method (KBM), initially developed by Keller [38] and then propagated by Cebeci [39], is now more commonly employed technique for addressing boundary layer problems in fluid dynamics. The KBM is demonstrated to be unconditionally stable and possesses second-order accuracy [40]. The outcome of this methodology arises from the subsequent steps:

- Initially, higher-order ODEs are transformed into first-order ODEs.
- The domain is discretized in the second stage, which is accomplished with the assistance of a central difference technique.
- Using the Newton''s approach, the non-linear ODEs are transformed into linear first-order ODEs in the third phase of the process.
- The last stage involves the utilization of the Block tri-diagonal elimination approach in order determine the linear ODEs.

To formally implement the proposed numerical technique, assume:

15 $$\left.\begin{array}{c}\kappa =f{\prime} ,\\ \tau =\kappa {\prime} ,\\ \varpi =\theta {\prime} ,\\ \omega =\phi {\prime} \mathrm{}.\end{array}\right\}$$

Now implement Eq. 15 and transformed the Eqs. 8–10.

16 $$\left.\begin{array}{l}\mathrm{(2}{\gamma }_{c}\eta +\mathrm{1)}\tau {\prime} +{\alpha }_{w}{\mathrm{(2}{\gamma }_{c}\eta +\mathrm{1)}}^{1.5}\tau {\prime} \tau +1.5{\gamma }_{c}{\alpha }_{w}\sqrt{\mathrm{(2}{\gamma }_{c}\eta +\mathrm{1)}}{(\tau )}^{2}-{P}_{2}M\,\sin ({\beta }_{a})\kappa \\ -{K}_{p}{\kappa }^{2}+{P}_{1}(\tau f-{\kappa }^{2}-{F}_{r}{\kappa }^{2}\mathrm{)=0,}\end{array}\right\}$$

17 $$\begin{array}{l}\left(\frac{4{R}_{d}}{3}+{P}_{4}\right)\mathrm{(2}{\gamma }_{c}\eta +\mathrm{1)}\varpi {\prime} +2\left(\frac{4{R}_{d}}{3}+{P}_{4}\right){\gamma }_{c}\varpi +{P}_{3}{P}_{r}\varpi f\mathrm{=0,}\end{array}$$

18 $$\mathrm{(2}{\gamma }_{c}\eta +\mathrm{1)}\omega {\prime} +2{\gamma }_{c}\omega +Sc\left(f\omega -{C}_{r}{(1+\tilde{\delta }\theta )}^{n}\exp \left(-\frac{{E}_{a}}{1+\tilde{\delta }\theta }\right)\phi \right)\mathrm{=0}.$$

We employ the central difference discretization on Eq. 15 and modify Eqs. 16, 17, and 18. This expression may be represented as: $\kappa {\prime} =\frac{{\kappa }_{i}-{\kappa }_{i-1}}{{h}_{i}}$ and $\tau =\frac{{\tau }_{i}+{\tau }_{i-1}}{2}$.

19 $$\left.\begin{array}{c}\frac{{\kappa }_{i-1}+{\kappa }_{i}}{2}=\frac{-{f}_{i-1}+{f}_{i}}{{h}_{i}},\\ \frac{{\tau }_{i-1}+{\tau }_{i}}{2}=\frac{-{\kappa }_{i-1}+{\kappa }_{i}}{{h}_{i}},\\ \frac{{\varpi }_{i-1}+{\varpi }_{i}}{2}=\frac{-{\theta }_{i-1}+{\theta }_{i}}{{h}_{i}},\\ \frac{{\omega }_{i-1}+{\omega }_{i}}{2}=\frac{-{\phi }_{i-1}+{\phi }_{i}}{{h}_{i}}\mathrm{}.\end{array}\right\}$$

20 $$\left.\begin{array}{c}\mathrm{(2}{\gamma }_{c}\eta +\mathrm{1)}\left(\frac{-{\tau }_{i-1}+{\tau }_{i}}{{h}_{i}}\right)+{\alpha }_{w}{\mathrm{(2}{\gamma }_{c}\eta +\mathrm{1)}}^{1.5}\left(\frac{-{\tau }_{i-1}+{\tau }_{i}}{{h}_{i}}\right)\left(\frac{{\tau }_{i-1}+{\tau }_{i}}{2}\right)\\ +1.5{\gamma }_{c}{\alpha }_{w}\sqrt{\mathrm{(2}{\gamma }_{c}\eta +\mathrm{1)}}{\left(\frac{{\tau }_{i-1}+{\tau }_{i}}{2}\right)}^{2}-{P}_{2}M\,\sin ({\beta }_{a})\left(\frac{{\kappa }_{i-1}+{\kappa }_{i}}{2}\right)\\ +{P}_{1}\left[\left(\frac{{\tau }_{i-1}+{\tau }_{i}}{2}\right)\left(\frac{{f}_{i-1}+{f}_{i}}{2}\right)-{\left(\frac{{\kappa }_{i-1}+{\kappa }_{i}}{2}\right)}^{2}-{F}_{r}{\left(\frac{{\kappa }_{i-1}+{\kappa }_{i}}{2}\right)}^{2}\right]\\ -{K}_{p}{\left(\frac{{\kappa }_{i-1}+{\kappa }_{i}}{2}\right)}^{2}\mathrm{=0,}\end{array}\right\}$$

21 $$\left.\begin{array}{c}\left(\frac{4{R}_{d}}{3}+{P}_{4}\right)\mathrm{(2}{\gamma }_{c}\eta +\mathrm{1)}\left(\frac{-{\varpi }_{i-1}+{\varpi }_{i}}{{h}_{i}}\right)+2\left(\frac{4{R}_{d}}{3}+{P}_{4}\right){\gamma }_{c}\left(\frac{{\varpi }_{i-1}+{\varpi }_{i}}{2}\right)\\ +{P}_{3}{P}_{r}\left(\frac{{\varpi }_{i-1}+{\varpi }_{i}}{2}\right)\left(\frac{{f}_{i-1}+{f}_{i}}{2}\right)\mathrm{=0,}\end{array}\right\}$$

22 $$\left.\begin{array}{c}\mathrm{(2}{\gamma }_{c}\eta +\mathrm{1)}\left(\frac{-{\omega }_{i-1}+{\omega }_{i}}{{h}_{i}}\right)+2{\gamma }_{c}\left(\frac{{\omega }_{i-1}+{\omega }_{i}}{2}\right)+Sc\left[\left(\frac{{f}_{i-1}+{f}_{i}}{2}\right)\left(\frac{{\omega }_{i-1}+{\omega }_{i}}{2}\right)\right]\\ -Sc{C}_{r}{\left[1+\tilde{\delta }\left(\frac{{\theta }_{i-1}+{\theta }_{i}}{2}\right)\right]}^{n}\exp \left(-\frac{{E}_{a}}{1+\tilde{\delta }\left(\frac{{\theta }_{i-1}+{\theta }_{i}}{2}\right)}\right)\left(\frac{{\phi }_{i-1}+{\phi }_{i}}{2}\right)\mathrm{=0}.\end{array}\right\}$$

We employ Newton''s technique to transform the discretized equations. This might be expressed by replacing ${\kappa }_{i}$ with $({\kappa }_{i}+\delta {\kappa }_{i})$ followed by reducing the modified equations by disregarding higher-order elements of δ.

23 $$\left.\begin{array}{r}\frac{\delta {\kappa }_{i-1}+{\kappa }_{i-1}+{\kappa }_{i}+\delta {\kappa }_{i}}{2}=\frac{-\delta {f}_{i-1}-{f}_{i-1}+{f}_{i}+\delta {f}_{i}}{{h}_{i}},\\ \frac{\delta {\tau }_{i-1}+{\tau }_{i-1}+{\tau }_{i}+\delta {\tau }_{i}}{2}=\frac{-\delta {\kappa }_{i-1}-{\kappa }_{i-1}+{\kappa }_{i}+\delta {\kappa }_{i}}{{h}_{i}},\\ \frac{\delta {\varpi }_{i-1}+{\varpi }_{i-1}+{\varpi }_{i}+\delta {\varpi }_{i}}{2}=\frac{-\delta {\theta }_{i-1}-{\theta }_{i-1}+{\theta }_{i}+\delta {\theta }_{i}}{{h}_{i}},\\ \frac{\delta {\omega }_{i-1}+{\omega }_{i-1}+{\omega }_{i}+\delta {\omega }_{i}}{2}=\frac{-\delta {\phi }_{i-1}-{\phi }_{i-1}+{\phi }_{i}+\delta {\phi }_{i}}{{h}_{i}}\mathrm{}.\end{array}\right\}$$

24 $$\left.\begin{array}{l}\mathrm{(2}{\gamma }_{c}\eta +\mathrm{1)}\left(\frac{-{\tau }_{i-1}-\delta {\tau }_{i-1}+{\tau }_{i}+\delta {\tau }_{i}}{{h}_{i}}\right)-{K}_{p}{\left(\frac{{\kappa }_{i-1}+\delta {\kappa }_{i-1}+{\kappa }_{i}+\delta {\kappa }_{i}}{2}\right)}^{2}\\ +1.5{\gamma }_{c}{\alpha }_{w}\sqrt{\mathrm{(2}{\gamma }_{c}\eta +\mathrm{1)}}{\left(\frac{{\tau }_{i-1}+\delta {\tau }_{i-1}+{\tau }_{i}+\delta {\tau }_{i}}{2}\right)}^{2}\\ -{P}_{2}M\,\sin ({\beta }_{a})\left(\frac{{\kappa }_{i-1}+\delta {\kappa }_{i-1}+{\kappa }_{i}+\delta {\kappa }_{i}}{2}\right)\\ +{P}_{1}\left[\left(\frac{{\tau }_{i-1}+\delta {\tau }_{i-1}+{\tau }_{i}+\delta {\tau }_{i}}{2}\right)\left(\frac{{f}_{i-1}+\delta {f}_{i-1}+{f}_{i}+\delta {f}_{i}}{2}\right)\right]\\ -{P}_{1}{\left(\frac{{\kappa }_{i-1}+\delta {\kappa }_{i-1}+{\kappa }_{i}+\delta {\kappa }_{i}}{2}\right)}^{2}-{P}_{1}{F}_{r}{\left(\frac{{\kappa }_{i-1}+\delta {\kappa }_{i-1}+{\kappa }_{i}+\delta {\kappa }_{i}}{2}\right)}^{2}\\ +{\alpha }_{w}{\mathrm{(2}{\gamma }_{c}\eta +\mathrm{1)}}^{1.5}\left(\frac{-{\tau }_{i-1}-\delta {\tau }_{i-1}+{\tau }_{i}+\delta {\tau }_{i}}{{h}_{i}}\right)\left(\frac{{\tau }_{i-1}+\delta {\tau }_{i-1}+{\tau }_{i}+\delta {\tau }_{i}}{2}\right)\mathrm{=0,}\end{array}\right\}$$

25 $$\left.\begin{array}{c}\left(\frac{4{R}_{d}}{3}+{P}_{4}\right)\mathrm{(2}{\gamma }_{c}\eta +\mathrm{1)}\left(\frac{-{\varpi }_{i-1}-\delta {\varpi }_{i-1}+{\varpi }_{i}+\delta {\varpi }_{i}}{{h}_{i}}\right)\\ +2\left(\frac{4{R}_{d}}{3}+{P}_{4}\right){\gamma }_{c}\left(\frac{{\varpi }_{i-1}+\delta {\varpi }_{i-1}+{\varpi }_{i}+\delta {\varpi }_{i}}{2}\right)\\ +{P}_{3}{P}_{r}\left(\frac{{\varpi }_{i-1}+\delta {\varpi }_{i-1}+{\varpi }_{i}+\delta {\varpi }_{i}}{2}\right)\left(\frac{{f}_{i-1}+\delta {f}_{i-1}+{f}_{i}+\delta {f}_{i}}{2}\right)\mathrm{=0,}\end{array}\right\}$$

26 $$\left.\begin{array}{c}\mathrm{(2}{\gamma }_{c}\eta +\mathrm{1)}\left(\frac{-{\omega }_{i-1}+\delta {\omega }_{i-1}+{\omega }_{i}+\delta {\omega }_{i}}{{h}_{i}}\right)+2{\gamma }_{c}\left(\frac{{\omega }_{i-1}+\delta {\omega }_{i-1}+{\omega }_{i}+\delta {\omega }_{i}}{2}\right)\\ +Sc\left[\left(\frac{{f}_{i-1}+\delta {f}_{i-1}+{f}_{i}+\delta {f}_{i}}{2}\right)\left(\frac{{\omega }_{i-1}+\delta {\omega }_{i-1}+{\omega }_{i}+\delta {\omega }_{i}}{2}\right)\right]\\ -Sc{C}_{r}{\left[1+\tilde{\delta }\left(\frac{{\theta }_{i-1}+\delta {\theta }_{i-1}+{\theta }_{i}+\delta {\theta }_{i}}{2}\right)\right]}^{n}\exp \left(-\frac{{E}_{a}}{1+\tilde{\delta }\left(\frac{{\theta }_{i-1}+\delta {\theta }_{i-1}+{\theta }_{i}+\delta {\theta }_{i}}{2}\right)}\right)\left(\frac{{\phi }_{i-1}+\delta {\phi }_{i-1}+{\phi }_{i}+\delta {\phi }_{i}}{2}\right)\mathrm{=0}.\end{array}\right\}$$

Now, generate the tri-diagonal matrix to get mathematical and graphical results.

27 $$\left(\begin{array}{ccccccc}[{\varLambda }_{1}] & [{\varUpsilon }_{1}] & & & & & \\ & [{\varLambda }_{2}] & [{\varUpsilon }_{2}] & & & & \\ & [{\varGamma }_{2}] & [{\varLambda }_{3}] & [{\varUpsilon }_{3}] & & & \\ & & [{\varGamma }_{3}] & [{\varLambda }_{4}] & [{\varUpsilon }_{4}] & & \\ & & & [{\varGamma }_{4}] & [{\varLambda }_{5}] & [{\varUpsilon }_{5}] & \\ & & & & [{\varGamma }_{5}] & [{\varLambda }_{6}] & [{\varUpsilon }_{6}]\\ & & & & & [{\varGamma }_{6}] & [{\varLambda }_{7}]\end{array}\right)\left(\begin{array}{c}{\rho }_{1}\\ {\rho }_{2}\\ {\rho }_{3}\\ {\rho }_{4}\\ {\rho }_{5}\\ {\rho }_{6}\\ {\rho }_{7}\end{array}\right)=\left(\begin{array}{c}{\varPi }_{1}\\ {\varPi }_{2}\\ {\varPi }_{3}\\ {\varPi }_{4}\\ {\varPi }_{5}\\ {\varPi }_{6}\\ {\varPi }_{7}\end{array}\right)$$

**Fig. 2:**
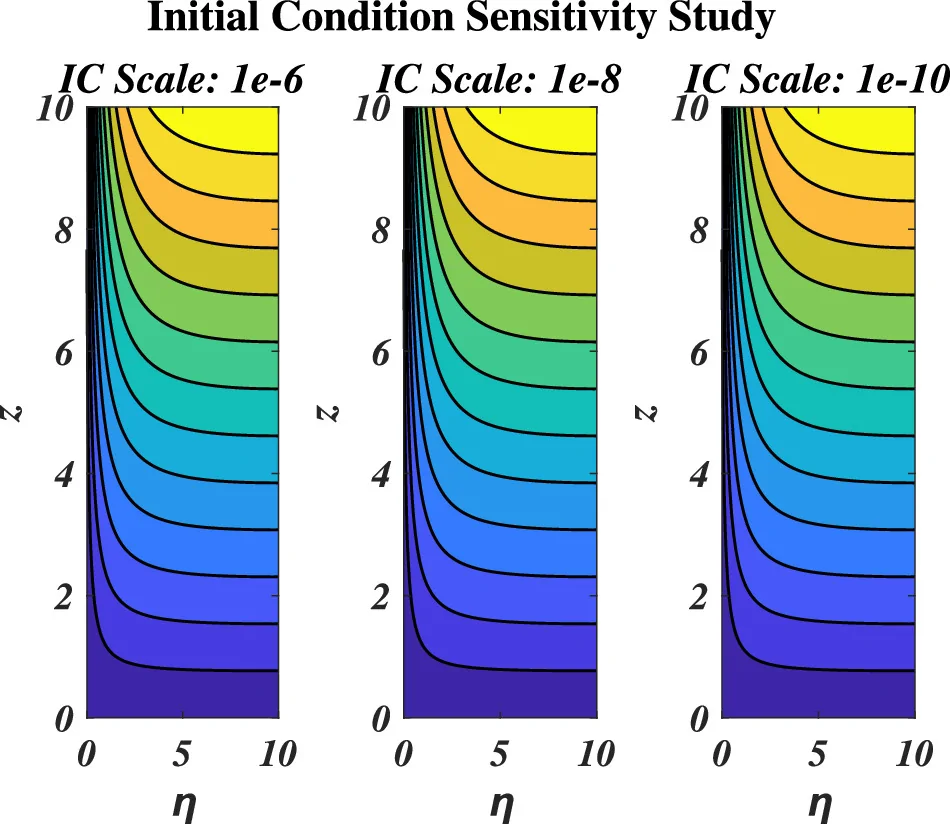
Sensitivity for initial conditions

**Fig. 3:**
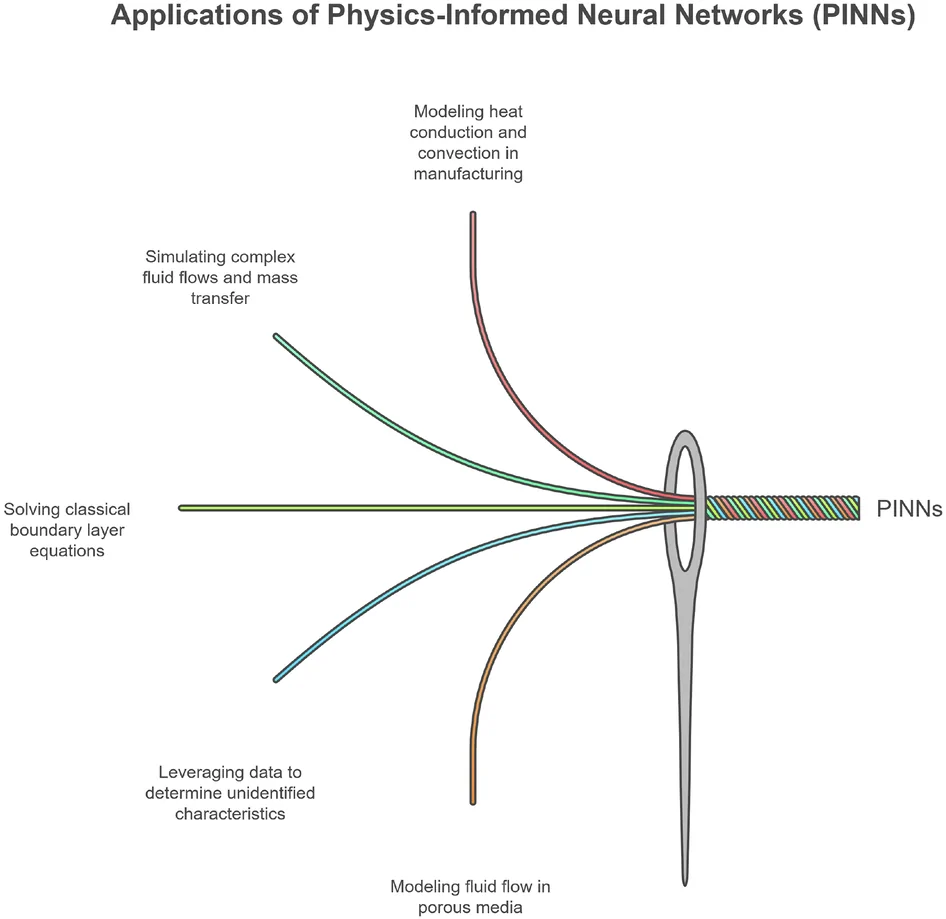
Applications of PINNs

The accuracy of the procedure depends on the sufficiency of the early forecasts, that are validated and shown in Fig. 2, whilst the precision of the methodology is determined by the choice of these preliminary estimates. Each subplot has a comparable pattern of curves, indicating that the outcome is fairly stable and not very reactive to the initial circumstances within the examined range. The Keller Box technique is used to provide accurate numerical solutions for the modified boundary-layer equations. This approach is especially effective for nonlinear boundary layer issues since it offers an efficient and second order precise finite difference scheme for interconnected nonlinear differential equations. Its inherent characteristics improve numerical stability for highly nonlinear and interconnected transport equations, while the semi-infinite computational domain may be efficiently managed by implementing a suitably extensive finite truncation boundary. The resultant high fidelity solutions are then used as benchmark data for the training and validation of the proposed PINN surrogate model. In 2019, Raissi et al. [41] coined the idea of physic informed neural networks (PINNs) to evaluate the PDEs. The potential uses of PINNs in numerous domains are shown in Fig. 3. Unlike conventional mesh-based methods that employed polynomials, PINN addresses its basis function through a singular deep neural network (DNN). Normally, the data points of the PDE and the boundary constraints (BC) are obtained from randomly selected sampling points within the computational space and at its boundary, correspondingly. The residuals of the established PDE and corresponding BCs are calculated utilizing the output of the DNN [34]. The loss function in PINNs is formulated by integrating the residuals of PDEs according to the initial and boundary constraints. Moreover, the objective of PINNs is to minimize the loss function while ensuring compliance with the starting and boundary constraints, as well as the residuals of the partial differential equations [42]. By implementing this approach, the network maintains the physical uniformity of the system throughout the solution procedure. Let us take the subsequent generic PDE to disclose the general implementation of PINNs:

28 $$\hat{N}x(t)=L(t),\,t\in \varOmega \mathrm{}.$$

29 $$x(t)=Q(t),\,t\in \partial \varOmega \mathrm{}.$$

Wherein $\hat{N}$ denotes the differential operator (linear or non-linear) and $\partial \varOmega$ represents the boundary of the computing domain Ω. x(t) is the solution of the PDE at the node t within the domain Ω, subject to the boundary value Q(t). Consistent with the PINN framework outlined by [33], a completely connected network that accepts x as inputs and generates ${U}_{N}(t)$ as responses can approach the solution x(t). The NN has multiple hidden layers, and the transmission of inputs $(Z=[{z}_{1},{z}_{2}\mathrm{..}.{z}_{j}])$ and outputs $(Y=[{y}_{1},{y}_{2}\mathrm{..}.{y}_{j}])$ for each hidden layer across the network is governed by:

30 $${y}_{i}={A}_{f}({z}_{j}{W}_{ji}+{B}_{j}\mathrm{)}.$$

where ${A}_{f},{B}_{j}$, and ${W}_{ji}$ denotes the activation function, bias, and weights of the network. The threshold settings can be optimized by minimizing a loss function defined as:

31 $$RES=RE{S}_{BC}+RE{S}_{PDE}$$

32 $$RE{S}_{PDE}=\frac{1}{{N}_{1}}\mathop{\sum }\limits_{j\mathrm{=1}}^{{N}_{1}}{(\hat{N}{U}_{N}({t}_{j})-L({t}_{j}))}^{2}$$

33 $$RE{S}_{BC}=\frac{1}{{N}_{2}}\mathop{\sum }\limits_{j\mathrm{=1}}^{{N}_{2}}{({U}_{N}({t}_{j})-Q({t}_{j}))}^{2}$$

Here, $RE{S}_{PDE},RE{S}_{BC},{N}_{1}$, ${N}_{2}$ signifies the residuals of the governing equations, residuals of the boundary conditions, quantity of data points for the governing and boundary conditions. The PINN framework employs automated differential within deep neural network algorithms to compute the derivatives of outcomes relative to inputs. Automatic differentiation is founded by the principle of chain rule, which enables the aggregation of the derivatives of discrete operations to get the derivative of the overall composition. Algorithm 1 presents the hybrid framework workflow for DNN-enriched PINNs evaluation and training In order to simulate complex multi-physics mechanisms, especially nanofluid flows, it is crucial to address non-linear ODEs and PDEs. While traditional mathematical methods like finite difference yield precise outcomes, they face challenges regarding scalability and real-time implementation. Developing machine learning frameworks like Physics-Informed Neural Networks (PINNs) offer a promising alternative, although they encounter challenges related to broad estimates and the need for complicated collocation points when addressing intricate shapes and coupled frameworks. By inherently depicting systematically discretized mechanisms as graphical representations, in which nodes serve distinct spatial or temporal points and edges capture local connections, GNNs address these limitations. GNNs are a category of deep neural networks intended to analyze grid-structured material by progressively refining node representations via message-passing across adjacent nodes. This framework is particularly advantageous for differential equations due to the intrinsic reliance of derivatives on neighborhood connections.

**Algorithm 1:**
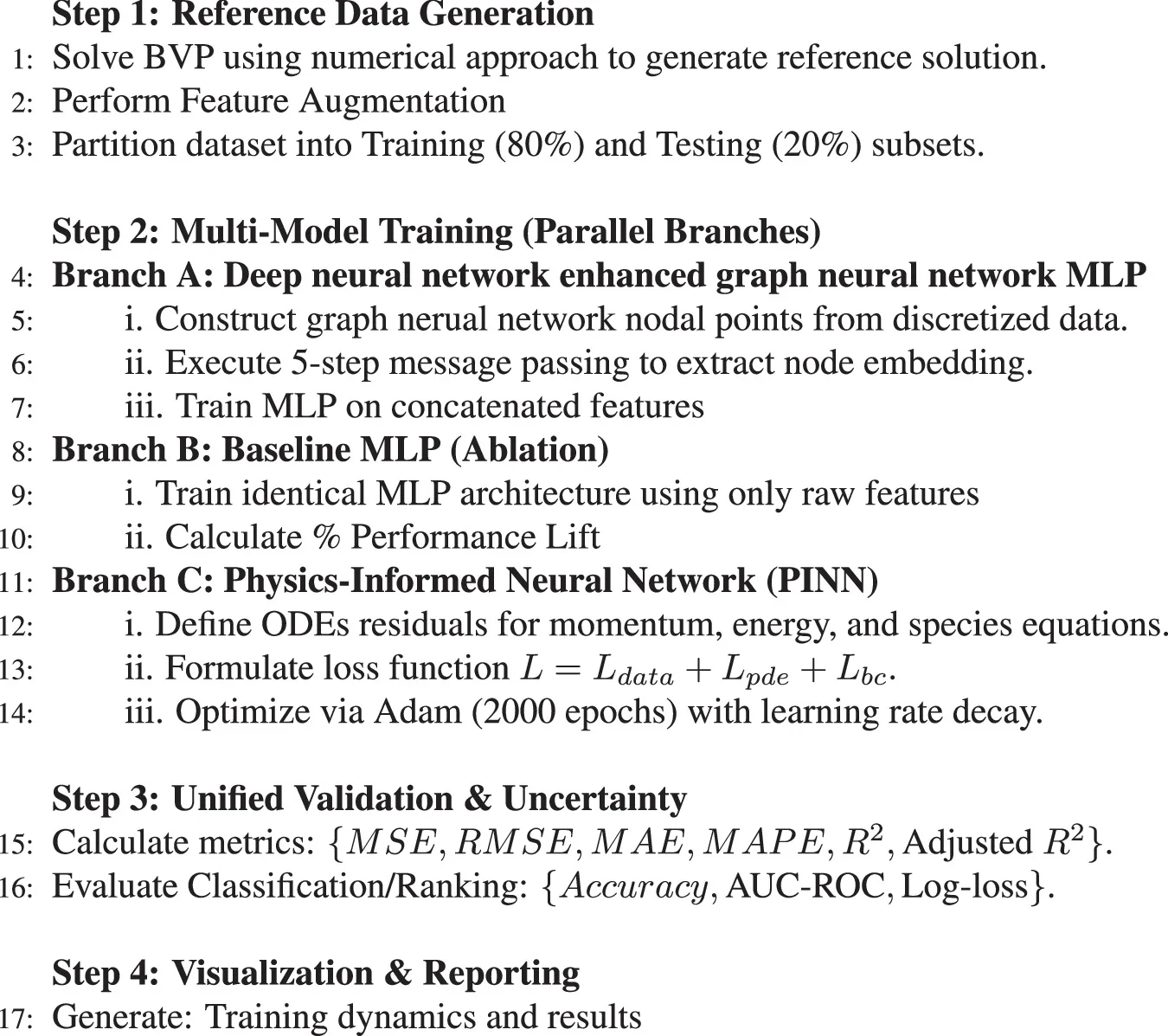
Comprehensive deep neural network enhanced graph neural network and PINNs training and simulation workflow

### DNN-enforced PINNs architecture

The model integrates an extensive array of variables and factors pertinent to the nanofluid system, encompassing fluid dynamics and nanoparticle attributes.

**Phase 1: GNN architecture and design**

- **Input layer**: We take $\eta \in \mathrm{[0,10]}$ as input layer.
- **Nodes**: We considered 1000 nodal points as discrete nodes for GNN.
- **Local Connectivity**: Every nodal point is attached to its nearest two neighboring edges.
- **Sparse Matrix**: An adjacency matrix (binary sparse matrix) has the form $A\in {R}^{1000\times 1000}\mathrm{}.$
- **Hidden states**: Every node j is composed of a state vector $({h}_{j})$ and the update rule is define as:

$${h}_{j}^{t+1}=\left[{W}_{rdm}\left\{{h}_{j}^{t},\frac{1}{|\hat{N}(j)|}\sum _{i\in \hat{N}(j)}{h}_{i}^{t}\right\}\right]tanh,$$

here, ${W}_{rdm}$ represents random weights simulated by MATLAB (randn). While tanh is the activation function. The implementation of tanh ensures that node update within range $[-\mathrm{1,1]}$.

**Fig. 4:**
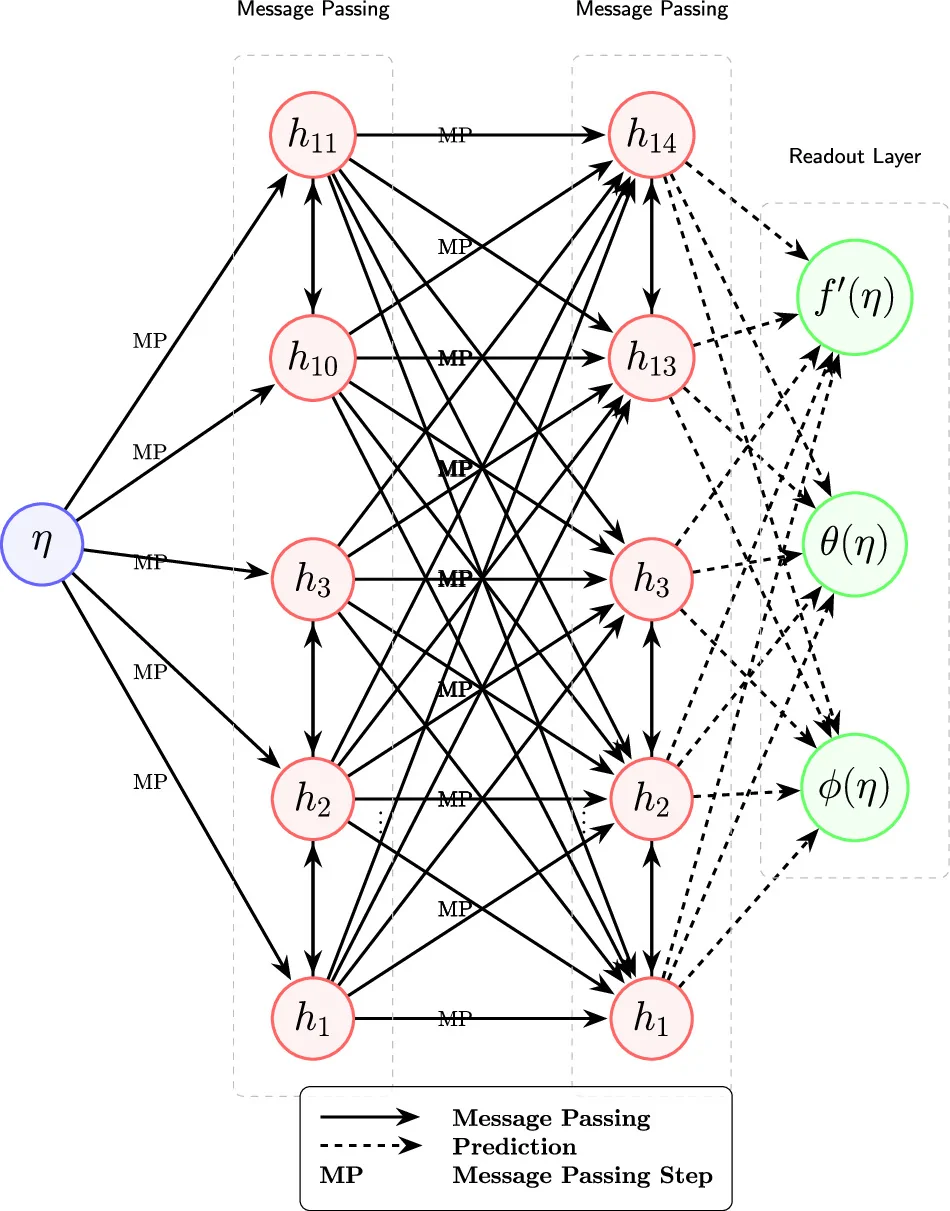
GNN architecture
- **Physics-informed residuals**: In this section we define the residual terms derived from governing ODEs, and we employed central difference approximation in this section, which are defined as:

- **Final prediction**: In the concluding prediction phase, the acquired node states are translated to the relevant physical field variables using a feed-forward neural network. The forecasting network has two fully interconnected hidden layers. The first hidden layer comprises 11 neurons using a Sigmoid activation function $sigm(z)=\frac{1}{1+\exp (-z)}$, whilst the subsequent hidden layer consists of 14 neurons employing the same Sigmoid activation. The output layer has three neurons that represent the dimensionless velocity, temperature, and concentration. A linear activation function is used in the output layer to facilitate the continuous ranges of the anticipated physical variables. Graphical outcomes of this data interpretation is presented in Fig. 4.

34 $$f{\prime} (\eta )\approx \frac{f({\eta }_{j+1})-f({\eta }_{j-1})}{2\varDelta \eta },\,f{\prime\prime} (\eta )\approx \frac{f({\eta }_{j+1})+f({\eta }_{j-1})-2f({\eta }_{j})}{\varDelta {\eta }^{2}}\mathrm{}.$$

Where $\varDelta (\eta \mathrm{)=0.01}$, and residuals are calculated utilizing these estimates at inner nodes.

**Phase 2: Physics compliance and training**

In this data generated for numerical evaluation for different governing parameters and for each data set contain 1000 nodal points for each solution (${f}^{\mathrm{''}}(\eta ),\theta (\eta )$, and $\phi (\eta )$). From Eq. 8, residual for momentum equation can be defined as:

35 $$\begin{array}{lll}{ {\mathcal R} }_{f} & =&{f{\prime\prime} {\prime} }_{{\rm{pred}}}+\frac{1}{\mathrm{(1}+2{\gamma }_{c}\eta )+{\alpha }_{w}{\mathrm{(1}+2{\gamma }_{c}\eta )}^{1.5}f{\prime\prime} }[1.5{\gamma }_{c}{\alpha }_{w}\sqrt{\mathrm{(2}{\gamma }_{c}\eta +\mathrm{1)}}{(f{\prime\prime} )}^{2}]\mathrm{..}.\\ && +\frac{1}{\mathrm{(1}+2{\gamma }_{c}\eta )+{\alpha }_{w}{\mathrm{(1}+2{\gamma }_{c}\eta )}^{1.5}f{\prime\prime} }[-{P}_{2}M\,\sin ({\beta }_{a})f{\prime} -{K}_{p}{f{\prime} }^{2}]\mathrm{..}.\\ && +\frac{1}{\mathrm{(1}+2{\gamma }_{c}\eta )+{\alpha }_{w}{\mathrm{(1}+2{\gamma }_{c}\eta )}^{1.5}f{\prime\prime} }[{P}_{1}(f{\prime\prime} f-{f{\prime} }^{2}-Fr{f{\prime} }^{2})]\end{array}$$

From Eq. 9, residual for energy equation is given as:

36 $${ {\mathcal R} }_{\theta }={\theta {\prime\prime} }_{{\rm{pred}}}+\frac{1}{\mathrm{(1}+2{\gamma }_{c}\eta )({P}_{4}+\frac{4}{3}{R}_{d})}\left[+2\left(\frac{4{R}_{d}}{3}+{P}_{4}\right){\gamma }_{c}\theta {\prime} +{P}_{3}{P}_{r}\theta {\prime} f\right]$$

Now from Eq. 10, residual for concentration of nanoparticles is provided as:

37 $${ {\mathcal R} }_{\phi }={\phi {\prime\prime} }_{{\rm{pred}}}+\frac{1}{1+2{\gamma }_{c}\eta }\left[2{\gamma }_{c}\phi {\prime} +Sc\left(f\phi {\prime} -{C}_{r}{(1+\tilde{\delta }\theta )}^{n}\exp \left(-\frac{{E}_{a}}{1+\tilde{\delta }\theta }\right)\phi \right)\right]$$

The residuals are included into the training phase via an integrated loss function which is define as:

**Total Loss**:

38 $${ {\mathcal L} }_{{\rm{total}}}=\lambda { {\mathcal L} }_{{\rm{physics}}}+{ {\mathcal L} }_{{\rm{data}}}\mathrm{}.$$

**Data Loss**:

39 $${ {\mathcal L} }_{{\rm{data}}}=\frac{1}{N}\mathop{\sum }\limits_{j\mathrm{=1}}^{N}{(\hat{f}\mathrm{''}({\eta }_{j})-{f{\prime} }_{{\rm{num}}}({\eta }_{j}))}^{2}+{(\hat{\theta }({\eta }_{j})-{\theta }_{{\rm{num}}}({\eta }_{j}))}^{2}+{(\hat{\phi }({\eta }_{j})-{\phi }_{{\rm{num}}}({\eta }_{j}))}^{2}\mathrm{}.$$

**Physics Loss**:

40 $${ {\mathcal L} }_{{\rm{physics}}}=\frac{1}{D}\mathop{\sum }\limits_{i\mathrm{=1}}^{D}({ {\mathcal R} }_{f}{({\eta }_{i})}^{2}+{ {\mathcal R} }_{\theta }{({\eta }_{i})}^{2}+{ {\mathcal R} }_{\phi }{({\eta }_{i})}^{2})\mathrm{}.$$

**Fig. 5:**
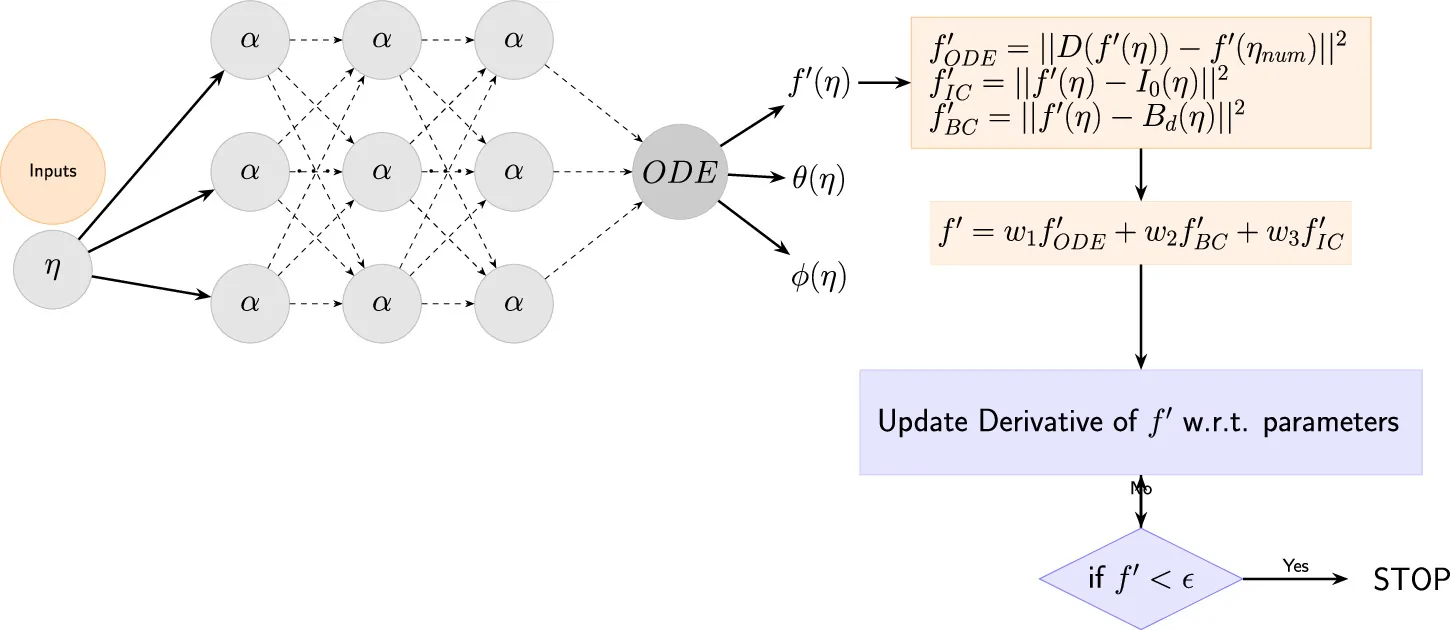
PINNs architecture for ${f}^{\mathrm{''}}$

**Fig. 6:**
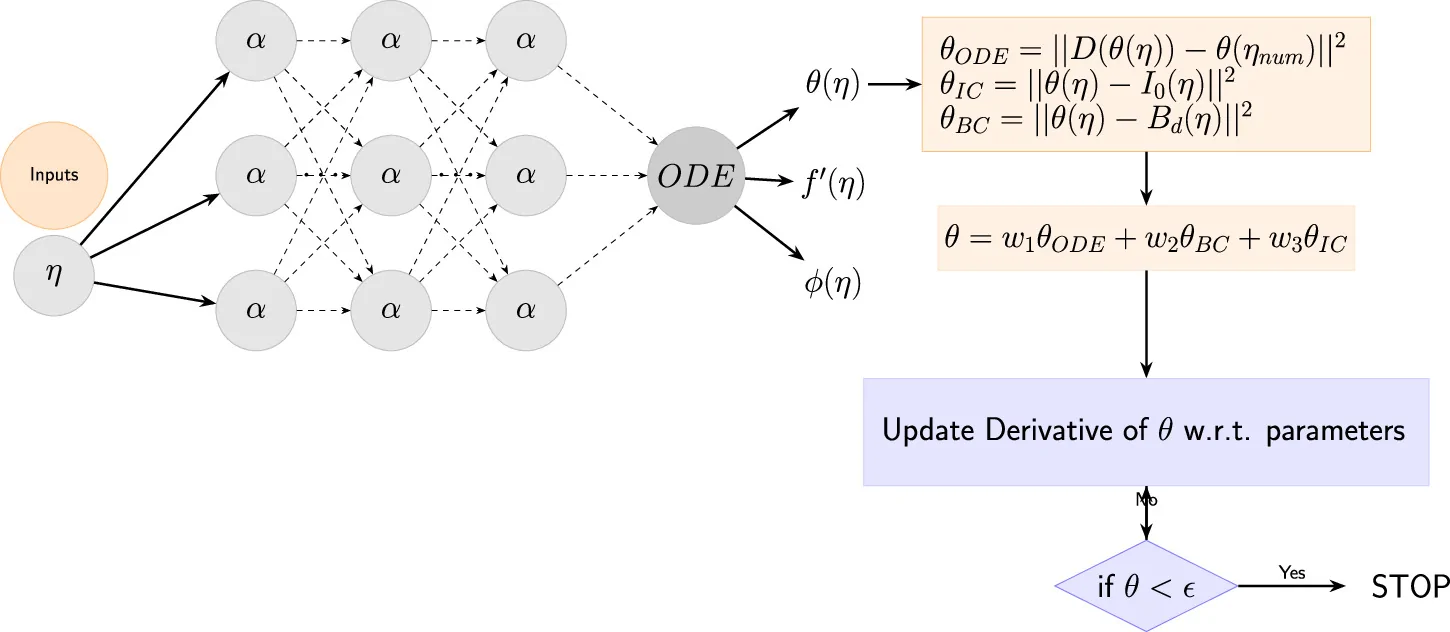
PINNs architecture for θ

**Fig. 7:**
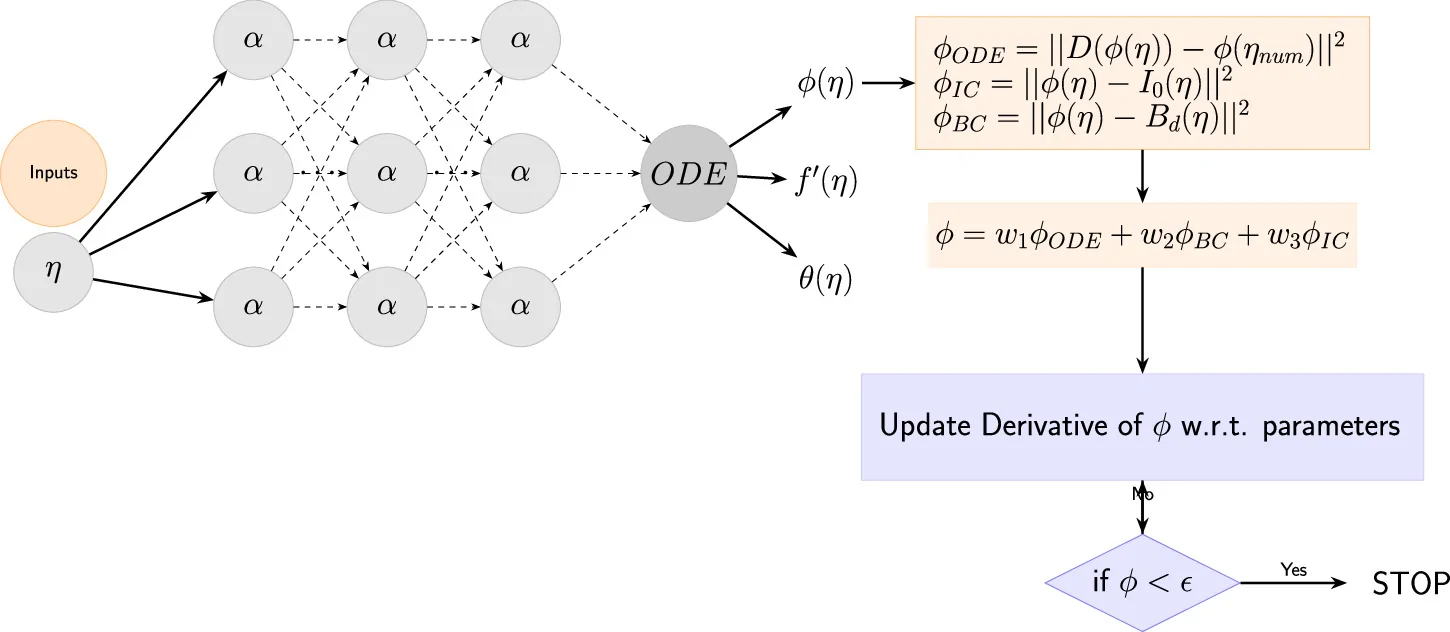
PINNs architecture for ϕ

**Fig. 8:**
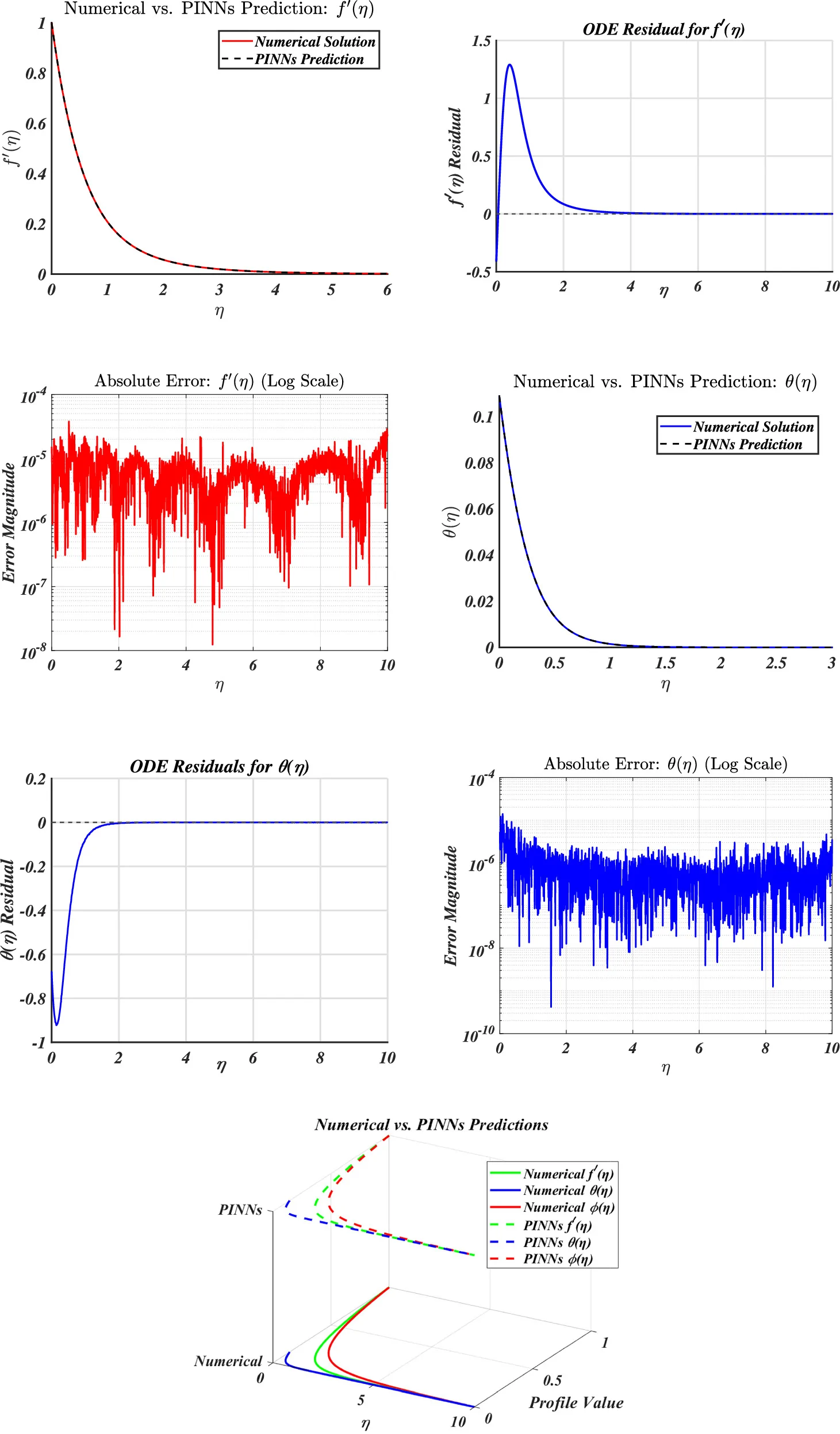
Numerical approach and PINNs results with residuals

**Fig. 9:**
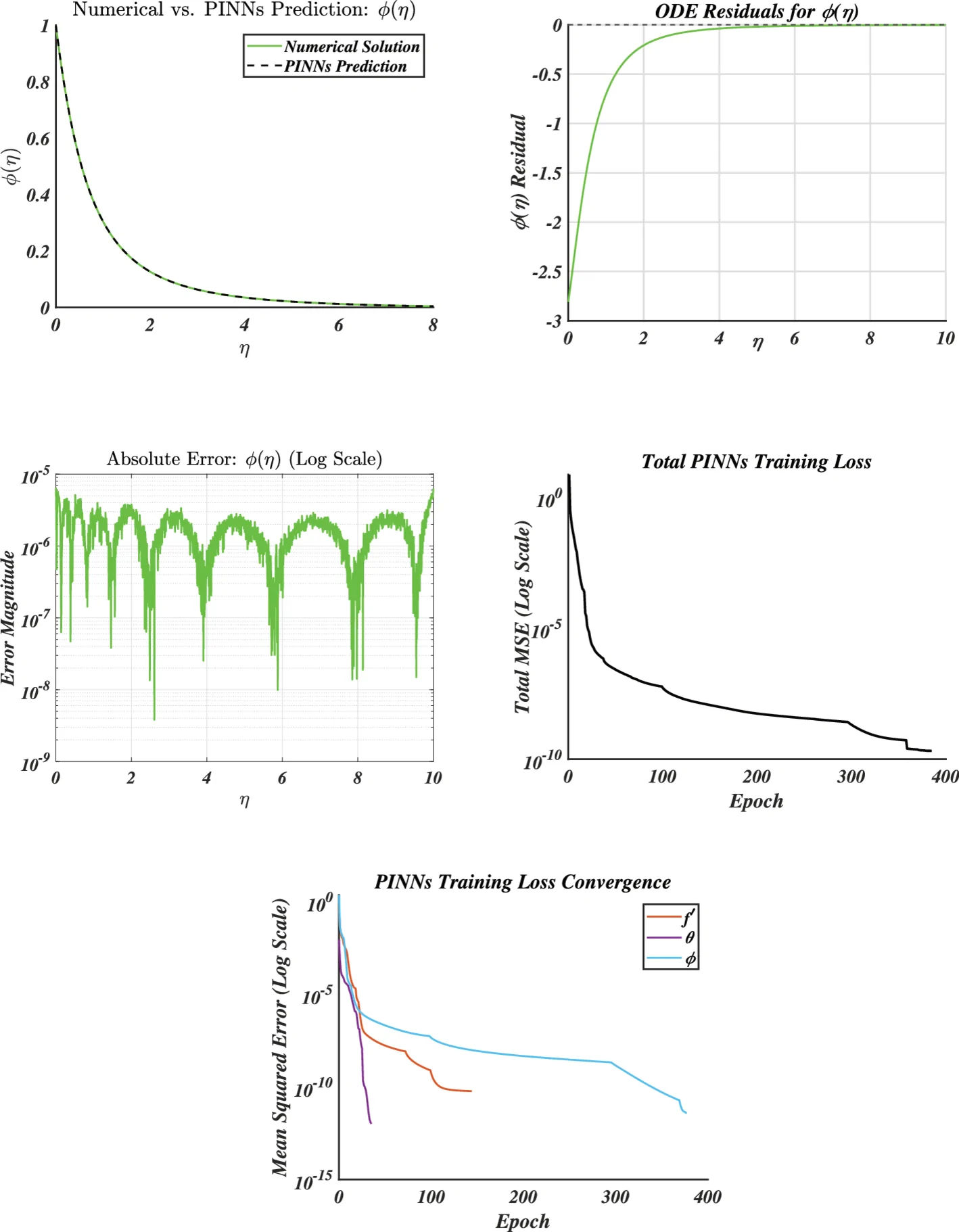
Numerical approach and PINNs results with residuals

**Fig. 10:**
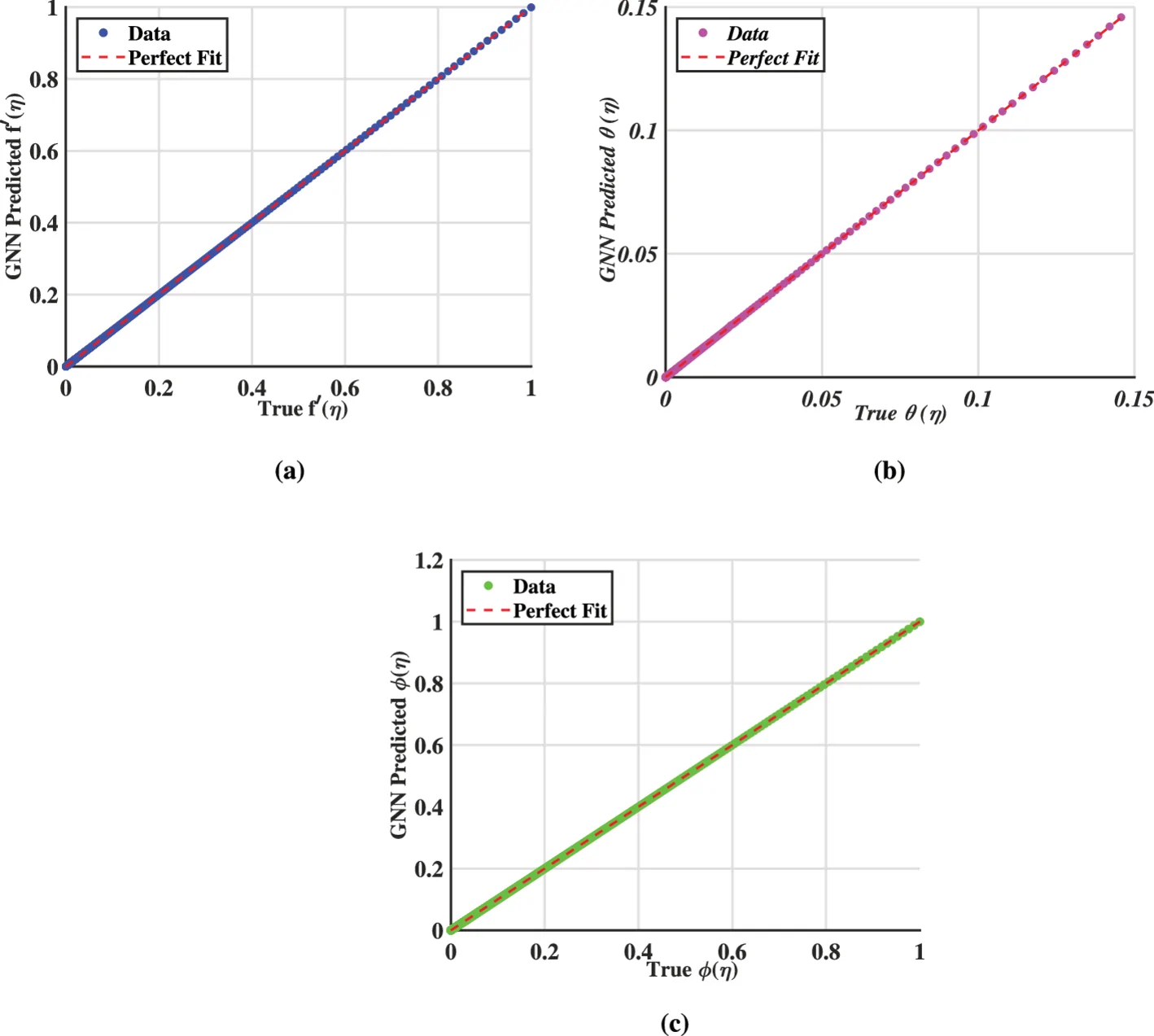
Comparative regression plots of the GNN-predicted versus true profiles for (**a**) dimensionless velocity gradient, **b** dimensionless temperature, and **c** dimensionless concentration

Figures 5, 6, and 7 PINNs architecture for different governing profiles. In this process, we employed the Levenberg-Marquardt algorithm with ${L}_{2}$ weight decay to optimize the training process. Figures 8 and 9 depicts the PINNs training parameters and as well as comparison with numerical scheme. It is evident from outcomes that PINNs mimic the numerical scheme outcomes. Figures 8 and 9 also illustrates the ODE residuals, absolute error (AE), and PINNs training loss function relative to the epoch number throughout the training phase. As the PINN training continues, the ODE residuals get closer to zero, showing that the training is effective at representing the system''s basic behavior. The y-axis, displayed on a logarithmic scale, illustrates the exponential reduction in loss as the number of epochs increases, signifying the model successful convergence. The total loss is around ${10}^{-9}$, indicating the network proficiency in precisely comprehending the fundamental physics of the problem. A total loss nearing zero signifies that the neural network has acquired a function that closely adheres to the fundamental physics of fluid flow. In PINNs, as opposed to just data-driven neural networks, the loss function has explicit physical significance. A converged loss indicates that the surrogate model is not just interpolating data but is instead influenced by physical principles and aligns with the mathematical framework of magnetized nanofluid flow around a porous cylinder. Figure 10 illustrate the regression analysis for GNN predicted and actual (numerical) values. The diagram demonstrates a robust correlation of data values with the ideal fit line, signifying the high precision and dependability of the GNN algorithm for forecasting these profiles.

### Global sensitivity analysis

A Global a sensitivity assessment (GSA) utilizing Sobol variance decomposition technique was performed to assess the relative impact of input factors on the thermal performance of the enclosure. The Sobol analysis systematically categorizes factors within an interconnected thermo-fluid system, emphasizing that the significance of parameters fluctuates according to the kind of response. The primary factors governing momentum are associated with flow resistance and transport, while thermal response is mostly affected by heat exchange and thermal diffusion. The concentration response highlights the importance of mass transport parameters in a ternary nanofluid. Disparities between first-order and total-order indices underscore the significance of parameter interactions. Consequently, the GSA delineates essential factors and facilitates prioritization, diminished of uncertainty, surrogate-model formulation, and multi-objective optimization.

The GSA accounts for non-linear interactions over the entire multi-modal input region, in contrast to local sensitivity approaches [43]. The total variance of the model output, L(Y), is decomposed into the partial variances attributable to individual model parameters and their interactions.

41 $$L(Y)=\mathop{\sum }\limits_{j\mathrm{=1}}^{k}{L}_{i}+\sum _{1\le j < l\le k}{V}_{jl}+\ldots +{L}_{\mathrm{1,2,}\ldots ,k},$$

where ${V}_{jl}$ illustrate variances. We employed total impact Sobol index ${S}_{Ti}$ as metric for comprehensive GSA [44]. Let us considers initial Sobol index $({S}_{i})$ and all superior-order interactions involving parameters $({X}_{i})$:

42 $${S}_{Ti}\mathrm{=1}-\frac{L(E[Y|{X}_{\sim i}])}{L(Y)}$$

The ${S}_{Ti}$ delineates involved parameters contribution to the result variation both independently and through how it interacts with all other factors. The convergence of metrics confirmed that the aforementioned physics-informed deep learning algorithm effectively maps the thermal response of the nanofluid. The research identifies the primary physical agents of the transmission of heat and indicates the potential for more precise adjustment of the hybrid nanofluid composition. The parameter space for the Sobol variance based decomposition was established by uniform sampling over the intervals: ${\gamma }_{c}\in \mathrm{[0.4,0.6],}{P}_{r}\in \mathrm{[18.24,27.36],}{\phi }_{1},{\phi }_{2}\in \mathrm{[0.020,0.030],}{B}_{i}\in \mathrm{[0.4,0.6]}$, and ${R}_{d}\in \mathrm{[0.4,0.6]}$. These limits reflect a $\pm \mathrm{20 \% }$ variation from the reference baseline (and physical limitations for volume fractions), guaranteeing that the analysis encompasses the local sensitivity framework while adhering to physical realistic values. Table 3 displays the first-order (${S}_{1}$) and total-order (${S}_{T}$) Sobol indices for the averaged responses of velocity, temperature, and concentration over the domain. The velocity is mostly influenced by ${\gamma }_{c}$ (${S}_{T}\mathrm{=0.487}$), followed by M (0.243), ${\alpha }_{w}$ (0.150), and ${F}_{r}$ (0.093), collectively accounting for about $\mathrm{97 \% }$ of the variation in response. These outcomes illustrates the explicit contributions of ${\gamma }_{c}$ in the geometric and nonlinear momentum components, M in the Lorentz force, and ${\alpha }_{w}$ and ${F}_{r}$ in the nonlinear rheological and porous-medium resistance phenomena. In terms of temperature, ${P}_{r}$ is the primary factor (${S}_{T}\mathrm{=0.717}$), followed by ${B}_{i}$ (0.189) and ${R}_{d}$ (0.101), aligning with their respective influences on thermal conduction, convective boundary conditions, and radiative heat exchange. The response to concentration is mostly governed by ${S}_{c}$ (${S}_{T}\mathrm{=0.931}$), with lesser influences from ${\gamma }_{c}$ (0.054) and ${E}_{a}$ (0.009), indicating ${S}_{c}$ direct role in species transport and the reaction term. The findings clearly identify the primary physical processes that regulate momentum, heat, and mass transmission.

**Table 3:** Sobol sensitivity indices for the velocity, temperature, and concentration responses

Parameter	Velocity		Temperature		Concentration	
	${S}_{1}$	${S}_{T}$	${S}_{1}$	${S}_{T}$	${S}_{1}$	${S}_{T}$
${\gamma }_{c}$	0.486	0.487	0.003	0.004	0.052	0.054
M	0.244	0.244	—	—	—	—
${\alpha }_{W}$	0.177	0.181	—	—	—	—
${F}_{r}$	0.093	0.093	—	—	—	—
${\beta }_{a}$	0.015	0.016	—	—	—	—
${P}_{r}$	—	—	0.714	0.717	—	—
${B}_{i}$	—	—	0.182	0.189	—	—
${R}_{d}$	—	—	0.093	0.101	—	—
${S}_{c}$	—	—	—	—	0.929	0.931
${E}_{a}$	—	—	—	—	0.015	0.017

## Results and discussion

**Fig. 11:**
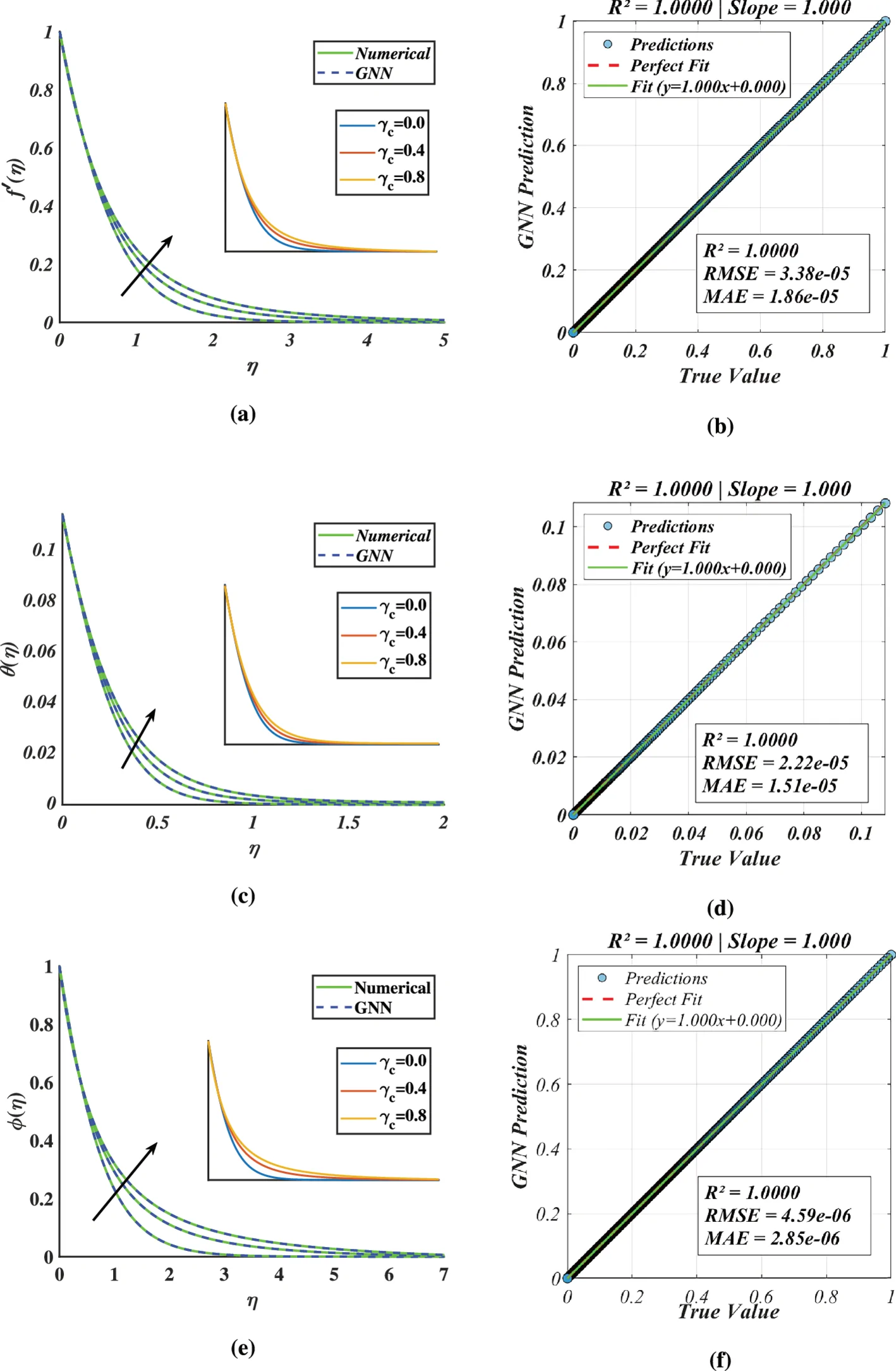
Validation of GNN predictions against numerical reference solution for varying curvature parameter: **a** dimensionless velocity profile, **b** regression analysis of velocity predictions with coefficient of determination, root mean square error (RMSE), and mean absolute error (MAE), **c** dimensionless temperature profile, **d** regression analysis of temperature predictions, **e** dimensionless concentration profile, and **f** regression analysis of concentration predictions

The present section examines the influence of physical variables on the hydrothermal characteristics of ternary nano-fluid. We supply pertinent visuals and data to elucidate the impacts of regulatory elements on fluid flow. Furthermore, we do an extensive comparative investigation of numerical approach and GNN for Williamson model ternary nanofluid fluid flow. Figure 11, and e demonstrate the impact of curvature parameter $({\gamma }_{c})$ for velocity $({f}^{\mathrm{''}}(\eta ))$, temperature$(\theta (\eta ))$, and concentration profile $(\phi (\eta ))$. The enhanced movement of fluids, heat transport, and mixing processes resulting from curvature account for the escalating trajectory of governing profiles with increasing ${\gamma }_{c}$. The observed enhancements in the respective profiles can be ascribed to increased centrifugal forces that elevate the velocity profile, improved convective heat transfer that amplifies the temperature profile, and more efficient mixing of nanoparticles that contributes to the upward trend of the concentration profile. Moreover, it is evident that GNN approach mimic numerical scheme successfully. Figure 11 also depicts the regression analysis for numerical and GNN predicted values. Different statistical parameters (RMSE, MAE, slope and ${R}^{2}$) are presented to evaluate the ML performance. Here ${R}^{2}$, RMSE, MAE, and slope represents the coefficient of determination, root mean square error, mean absolute error, and the slope of linear regression model, respectively. Mathematical expression for these parameters are:

43 $$\begin{array}{ll}{R}^{2}= & 1-\frac{{\rm{Sum\:of\:squares\:of\:residuals}}}{{\rm{total\:sum\:of\:squares}}},\,MAE=\frac{1}{N}\mathop{\sum }\limits_{j\mathrm{=1}}^{N}|{Y}_{j}-{\hat{Y}}_{j}|,\\ & RMSE=\sqrt{\frac{1}{N}\mathop{\sum }\limits_{j\mathrm{=1}}^{N}{({Y}_{j}-{\hat{Y}}_{j})}^{2}}\mathrm{}.\end{array}$$

**Fig. 12:**
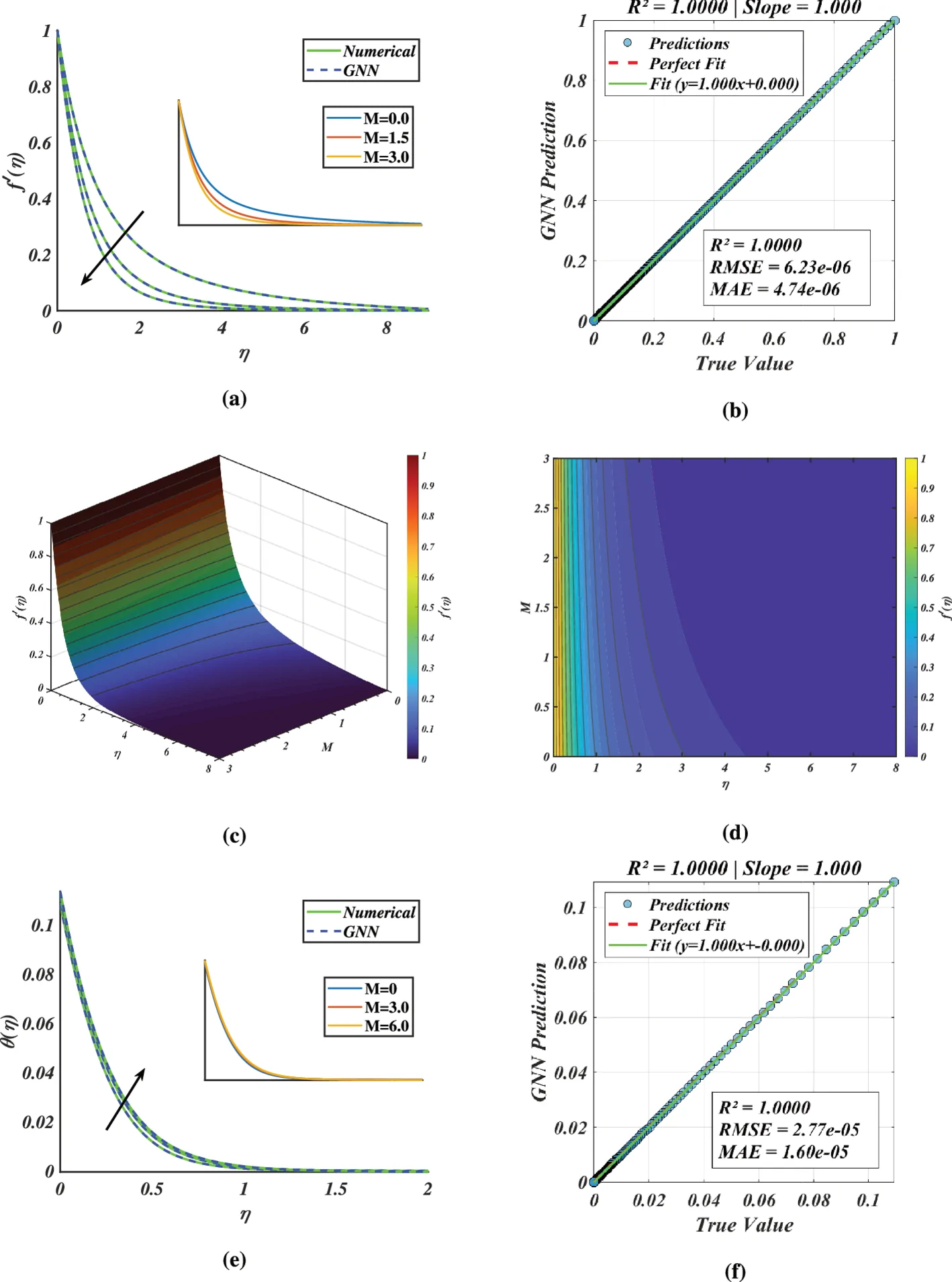
Validation of GNN predictions against numerical reference solution for varying magnetic parameter: **a** dimensionless velocity profile, **b** regression analysis of velocity predictions with coefficient of determination, root mean square error (RMSE), and mean absolute error (MAE), **c** surface plot of magnetic parameter for velocity profile, **d** contour plot of magnetic parameter for velocity profile, **e** dimensionless temperature profile, and **f** regression analysis of temperature predictions

**Fig. 13:**
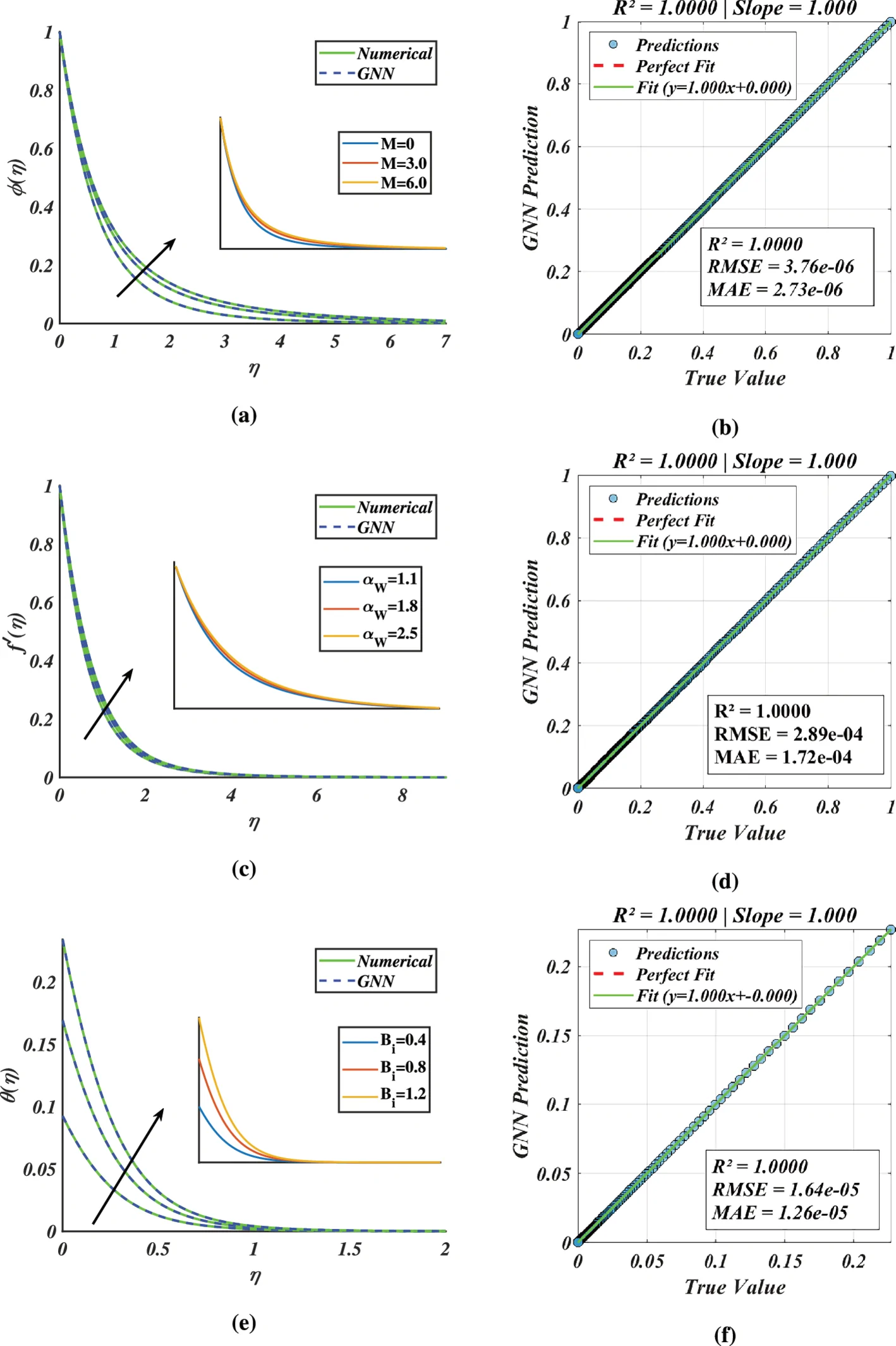
Validation of GNN predictions against numerical reference solution for varying magnetic, Williamson fluid parameter, and Biot number: **a** dimensionless concentration profile for M, **b** regression analysis of concentration predictions with coefficient of determination, root mean square error (RMSE), and mean absolute error (MAE) for M, **c** dimensionless velocity profile for ${\alpha }_{W}$, **d** regression analysis of velocity predictions for ${\alpha }_{W}$, **e** dimensionless temperature profile for ${B}_{i}$, and **f** regression analysis of temperature prediction for ${B}_{i}$

**Fig. 14:**
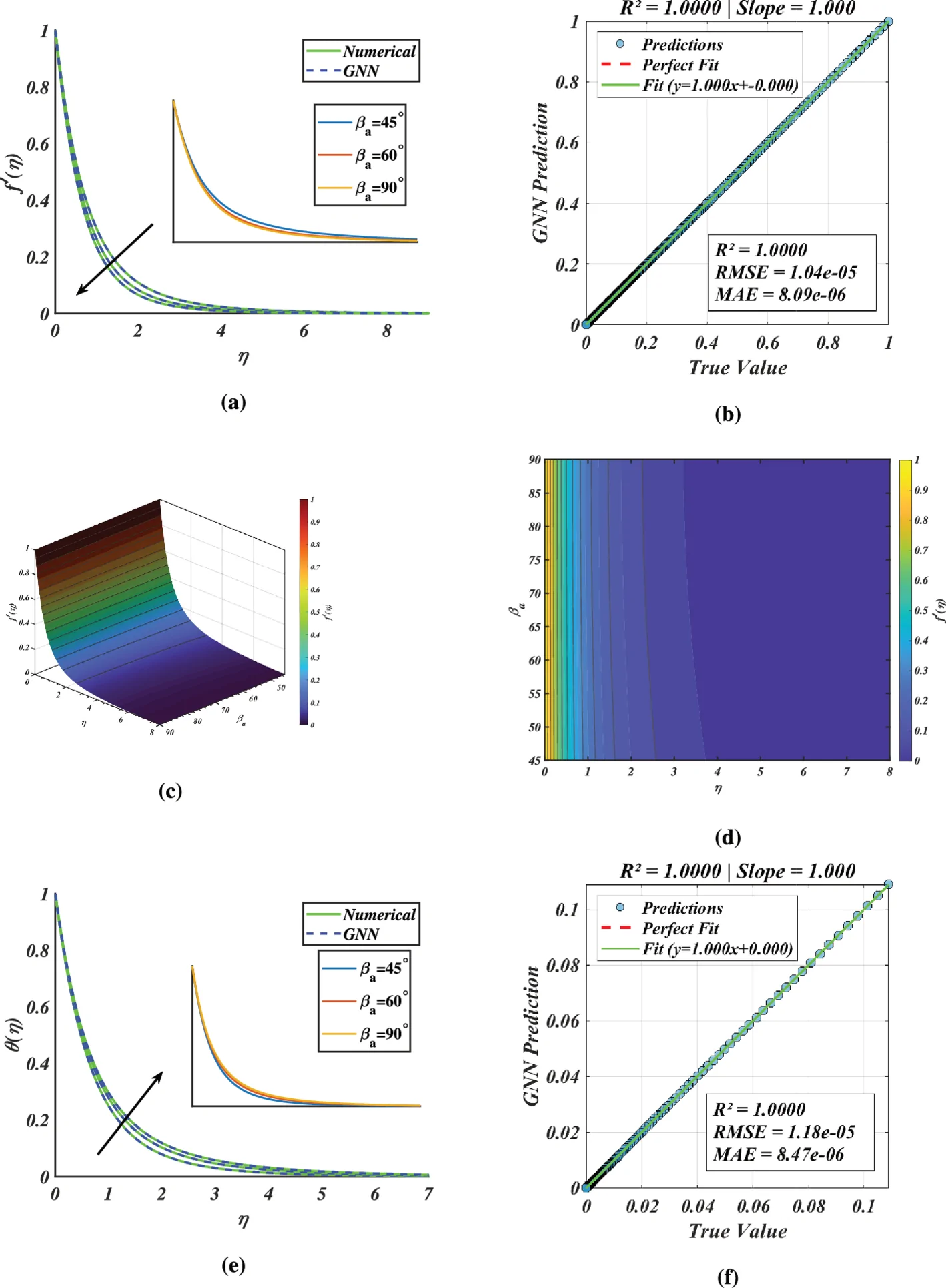
Validation of GNN predictions against numerical reference solution for varying inclination angle $({\beta }_{a})$: **a** dimensionless velocity profile, **b** regression analysis of velocity predictions with coefficient of determination, root mean square error (RMSE), and mean absolute error (MAE), **c** surface plot of ${\beta }_{a}$ for velocity profile, **d** contour plot of ${\beta }_{a}$ for velocity profile, **e** dimensionless temperature profile, and **f** regression analysis of temperature predictions

**Fig. 15:**
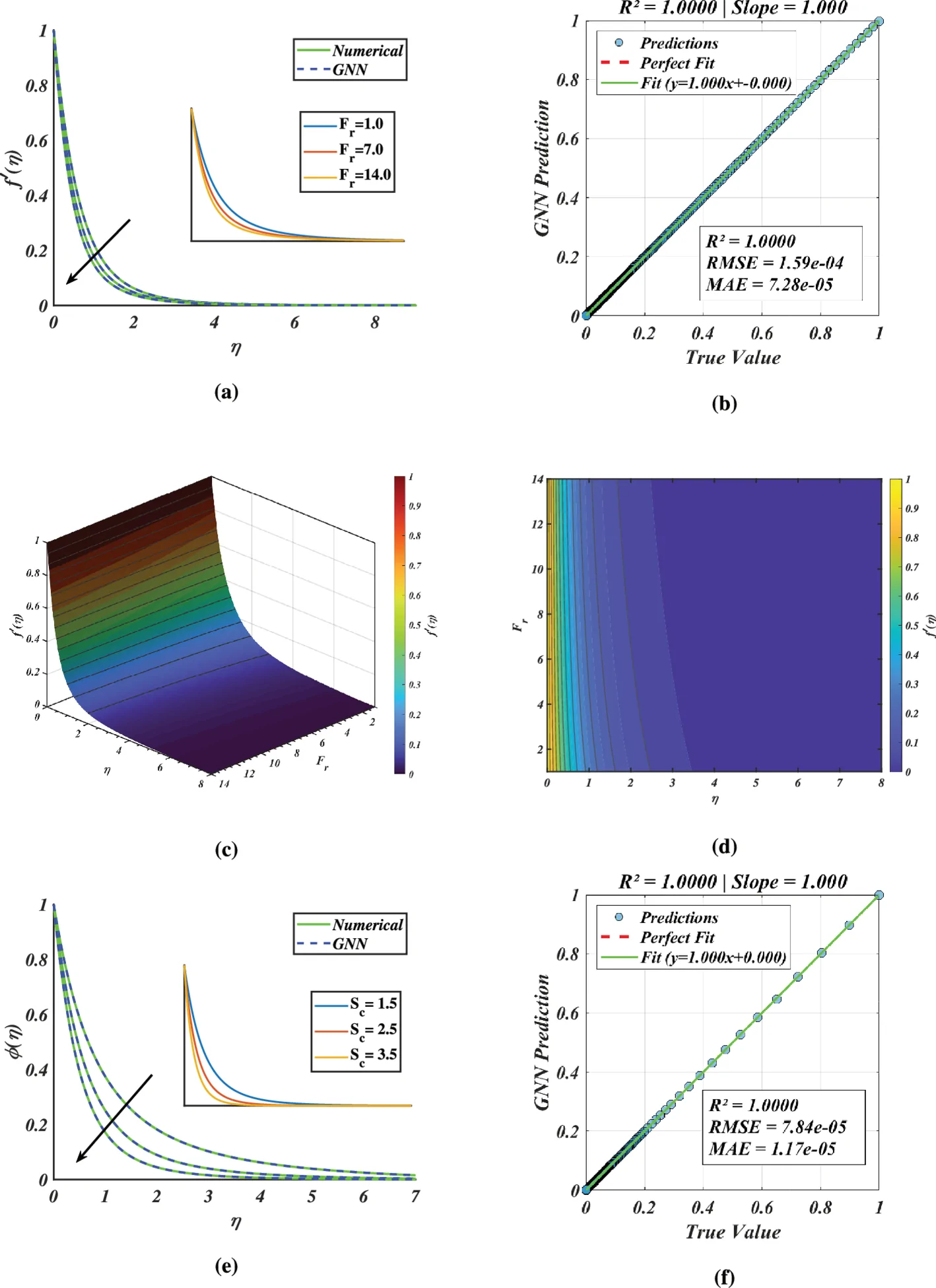
Validation of GNN predictions against numerical reference solution for varying Forchhieimer number $({F}_{r})$, and Schmidt number $({S}_{c})$: **a** dimensionless velocity profile for ${F}_{r}$, **b** regression analysis of velocity predictions with coefficient of determination, root mean square error (RMSE), and mean absolute error (MAE) for ${F}_{r}$, **c** surface plot of ${F}_{r}$ for velocity profile, **d** contour plot of ${F}_{r}$ for velocity profile, **e** dimensionless concentration profile for ${S}_{c}$, and **f** regression analysis of concentration predictions

**Fig. 16:**
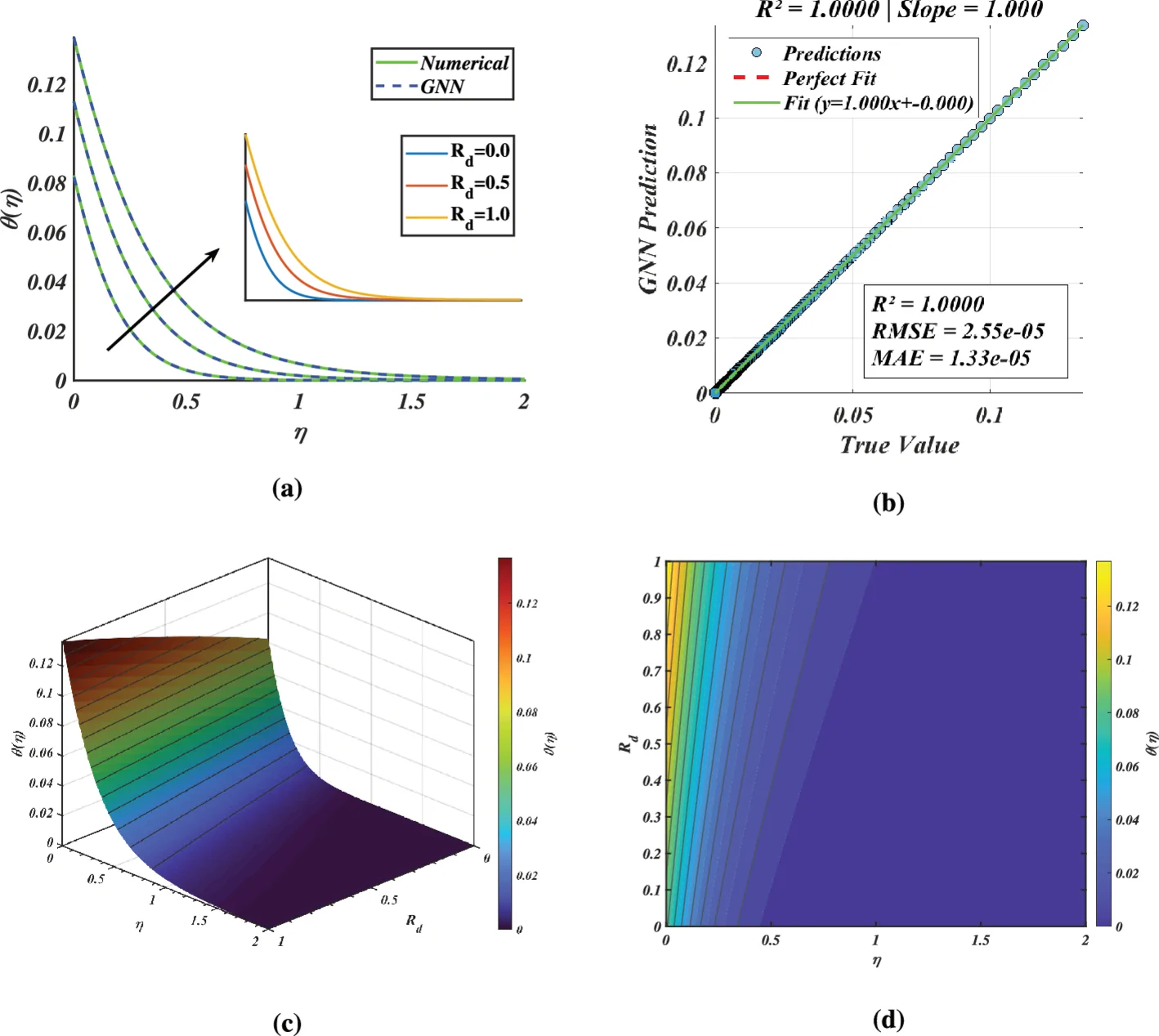
Validation of GNN predictions against numerical reference solution for varying radiation parameter: **a** dimensionless temperature profile, **b** regression analysis of temperature predictions with coefficient of determination, root mean square error (RMSE), and mean absolute error (MAE), **c** surface plot of ${R}_{d}$ for temperature profile, **d** contour plot of ${R}_{d}$ for temperature profile

**Fig. 17:**
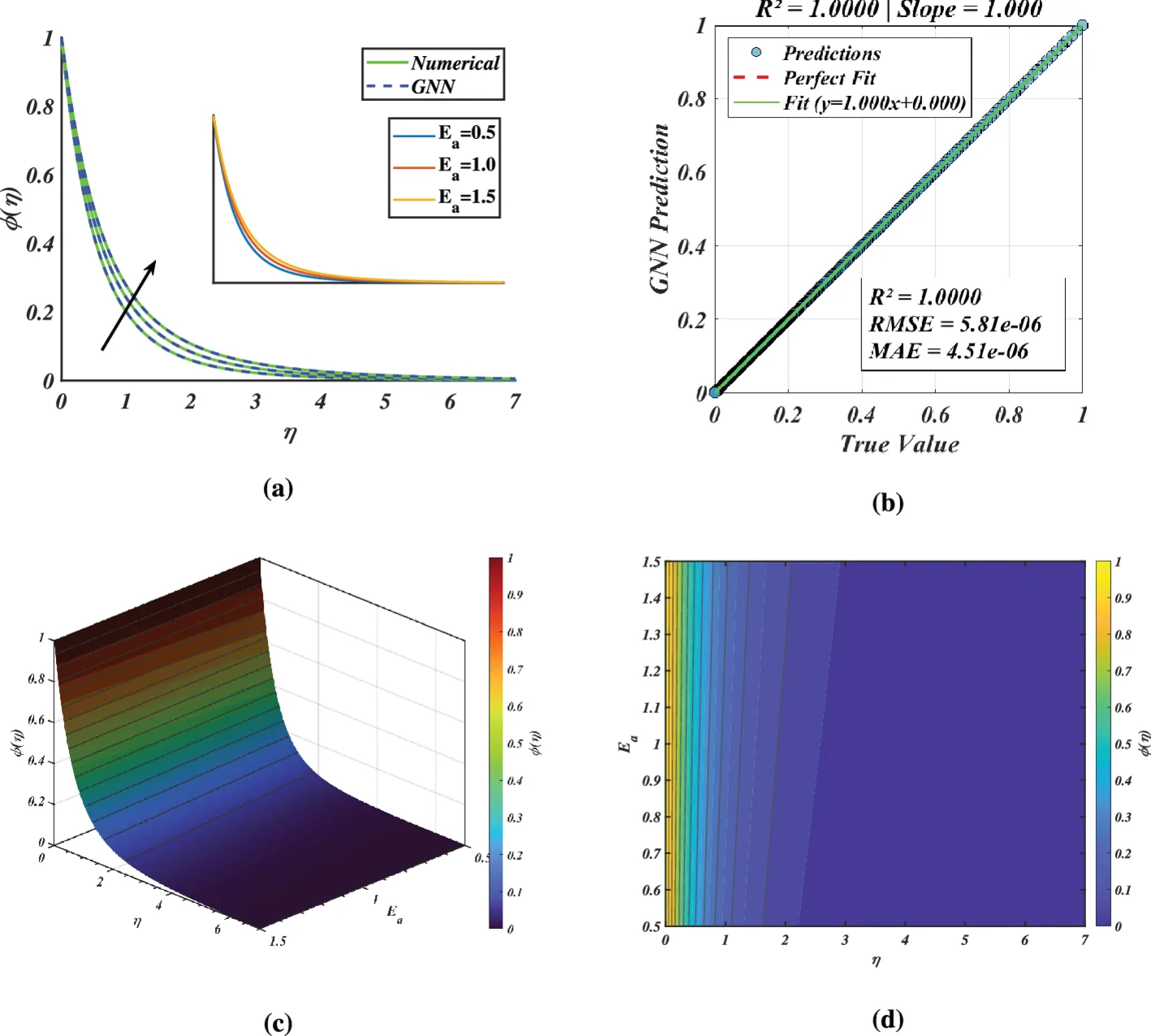
Validation of GNN predictions against numerical reference solution for varying activation energy parameter: **a** dimensionless concentration profile, **b** regression analysis of concentration predictions with coefficient of determination, root mean square error (RMSE), and mean absolute error (MAE), **c** surface plot of ${E}_{a}$ for concentration profile, **d** contour plot of ${E}_{a}$ for concentration profile

**Fig. 18:**
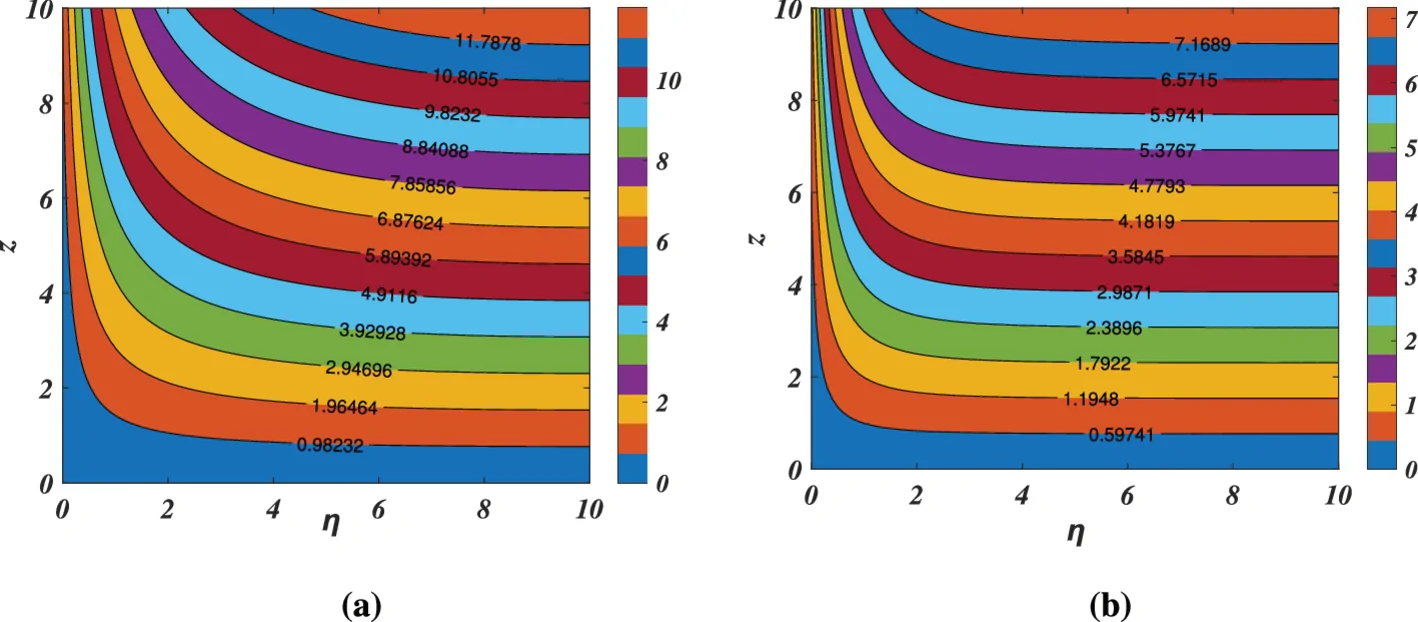
Streamlines

The values of ${R}^{2}$ ranges between 1 and 0. If the value of ${R}^{2}\mathrm{=0}$ it means there is no relationship among variables, while ${R}^{2}\mathrm{=1}$ indicate a strong relationship (perfect fit). The standard deviation of residuals is quantified by RMSE. Lower values of RMSE depict good fit. Mean scale of errors of predictions is determines by MAE. The steepness or gradient of regression line is denoted by slope. In ideal and perfect scenarios, its value must equal to 1. Outcomes indicates that GNN machine learning model projections, ${R}^{2}\mathrm{=1}$, lower values of RMSE and MAE, are exceptionally precise and dependable for the specified purpose. Figure 12, and Fig. 13 illustrates the influence of the magnetic parameter (M) on the velocity, heat, and concentration profiles, with GNN statistical metrics, respectively. Results are presented for both numerical and GNN approach. An increase in M causes an intensified Lorentz effect, which hinders the movement of fluid components, resulting in a diminished velocity profile $({f}^{\mathrm{''}}(\eta ))$. The Lorentz force influencing the fluid flow generates kinetic energy, resulting in elevated temperature and concentration profiles, reflecting the patterns noted in the experimental study of MHD nanofluid flow by Ammar et al. [45], thereby validating the present research. Statistical parameter associated with ML model for M are also presented in Fig. 12. For velocity, temperature and concentration profile the values of ${R}^{2}$ equals 1, which illustrates that numerical outcomes perfectly matches the predicted values by GNN. RMSE values of $6.23e-\mathrm{06,2.77}e-05$, and 3.76e−06 versus ${f}^{\mathrm{''}}(\eta ),\theta (\eta )$, and $\phi (\eta )$ for M demonstrate good precision for prediction. Figure 13 are drawn to elucidates the impact of Williamson fluid parameter $({\alpha }_{W})$, and the Biot number $({B}_{i})$ against velocity and temperature distribution of ternary nanofluid by GNN and numerical approach. The ${\alpha }_{W}$ physically induces an augmented viscoelastic characteristic that improves momentum transfer in the ternary nanofluid, resulting in an elevated velocity profile. An increase in temperature and the associated boundary layer is essential for enhanced heat transfer due to the ${B}_{i}$. To illustrate the inclination angle of applied magnetic field with statistical metrics for GNN, Fig. 14 is constructed. Rising values of ${\beta }_{a}$ slow down the ${f}^{\mathrm{''}}(\eta )$, and uplift $\theta (\eta )$, respectively. The ${\beta }_{a}$ provide a gravitational force aligned with the fluid movement, enhancing the convective heat transfer rate and raising the temperature distribution as seen in the given graphical outcome. Moreover, this gravitational force also induces velocity of fluid flow and facilitate the mixing and uniform distribution of temperature. Statistical parameter of ML training depict a good agreement between GNN and numerical approach while computing these parameters. Figure 15 is drawn to show the impacts of Forchheimer number $({F}_{r})$, and Schmidt number $({S}_{c})$ for velocity and concentration profiles including performance metrics. Increased ${F}_{r}$ values indicate that the velocity profile has a downward tendency due to heightened frictional forces. Enhancing ${S}_{c}$ reduces mass transfer efficiency, resulting in a more homogeneous concentration distribution and a decline in $\phi (\eta )$. The thermal boundary layer thickens with a spike in the ${R}_{d}$ frequency due to enhanced heat transmission within the fluid, as depicted in Fig. 16. One possibility that may elucidate this trend is that thermal radiation converts into electromagnetic energy. Moreover, Fig. 16 demonstrate the effectiveness of GNN approach with numerical scheme. For ${F}_{r},{B}_{i}$, and ${R}_{d}$, the coefficients of determination equal 1, indicating that the numerical outputs perfectly align with the projected values using GNN. RMSE values of 1.59e−04, 1.64e−05, and 2.55e−05 for these corresponding parameters indicate high predictive accuracy of ML model. Lower values of MAE values indicate an excellent fit. To illustrate the effects of activation energy $({E}_{a})$ against the concentration profile $(\phi (\eta ))$, Fig. 17 is sketched. The diagram illustrates that the $\phi (\eta )$ grows with an elevation in the ${E}_{a}$. Increasing the ${E}_{a}$ reduces the speed of the chemical reaction, leading to a higher concentration of reactants and hence an increase in $\phi (\eta )$. The value of ${R}^{2}$ for ${E}_{a}$, and ${S}_{c}$ equal 1, signifying that the numerical outputs are in perfect concordance with the predicted values derived by GNN. The RMSE values of 5.81e−06, and 7.84e−05 for the respective parameters demonstrate the outstanding prediction accuracy of the machine learning model. Moreover, reduced MAE values signify a superior match. Figure 18 depicts the streamlines for investigated problem. Due the lack of a magnetic force (Fig. 18 when $M\mathrm{=0}$), a conventional fluid boundary layer is characterized by uniform, constant streamlines with a boundary layer typically increases in thickness as it progresses downstream. But a Lorentz force induces a deceleration of the liquid in the context of a magnetic field (Fig. 18 when $M\mathrm{=3}$), thus lowering the top velocity to around 7.17. As one approaches the outer layer, the streamlines become more horizontal. This illustrates how the magnetic field diminishes the velocity slope, curtails the growth of the boundary layer, and regulates the motion of the fluid. Table 4 offers a succinct overview of the neural network training efficacy, detailing the MAE and RMSE metrics for the predicted velocity, temperature, and concentration profiles, thereby illustrating the model prediction precision and convergence. Table 5 presents a comparison of our findings with the Nisar et al. [46], and Shah et al. [36] for certain limiting thresholds of the standard parameter.When compared to the conclusions of earlier research, our results emerge to be consistent.

**Table 4:** Overall statistical performance of the neural network for different physical parameters and response profiles

Parameter	Profile	MAE	RMSE
${\gamma }_{c}$	Velocity profile	1.86e–05	3.38e–05
${\gamma }_{c}$	Temperature profile	1.51e–05	2.22e–05
${\gamma }_{c}$	Concentration profile	2.85e–06	4.59e–06
M	Velocity profile	4.74e–06	6.23e–06
M	Temperature profile	1.60e–05	2.77e–05
M	Concentration profile	2.73e–06	3.76e–06
${\alpha }_{W}$	Velocity profile	1.72e–04	2.89e–04
${B}_{i}$	Temperature profile	1.26e–05	1.64e–05
${\beta }_{a}$	Velocity profile	8.09e–06	1.04e05
${\beta }_{a}$	Temperature profile	8.47e–06	1.18e–05
${F}_{r}$	Velocity profile	7.28e–05	1.59e–04
${S}_{c}$	Concentration profile	1.17e–05	7.84e–05
${R}_{d}$	Temperature profile	1.33e–05	2.55e–05
${E}_{a}$	Concentration profile	4.51e–06	5.81e–06

**Table 5:** Analysis of $f{\prime\prime} \mathrm{(0)}$ across different values of M, while keeping all other parameters at zero

M	Nisar et al. [46]	Shah et al. [36]	Present Outcomes
0.0	−1.00000	−0.999305	−0.99860
1.0	−1.4137	−1.414213	−1.41390
5.0	−2.4495	−2.449490	−2.44905
10.0	−3.3166	−3.316627	−3.31589

## Conclusion

The study introduces a novel, and accurate physics-informed machine learning method for examining Williamson model ternary nanofluid over a stretching cylinder. The present investigation is motivated by the absence of recorded attempts to address coupled fluid flow systems using stochastic methods. The proposed hybrid technique utilizes physics-informed neural networks optimized by deep neural network. This hybrid technique uses deep learning capabilities by directly incorporating fundamental physical principles into the loss function, enabling the model to generate responses that are data-driven and physics-constrained. This approach is particularly advantageous for complex systems, as it ensures that the projected results conform to established physical laws. Important concluding remarks are given as:

- The findings indicate that the suggested PINN can successfully replicate the nonlinear transport characteristics while offering a computationally economical substitute for repetitive numerical simulations in parametric forecasting.
- The temperature profile, concentration, and fluid velocity increase with the strengthening of the curvature variable.
- The findings from the GSA reveal that the curvature parameter, along with nanoparticle concentration, are the primary factors influencing the flow rate. The fluid temperature exhibits a high sensitivity to the Biot number, nanoparticle concentration, and thermal radiation.
- The flow velocity escalates with the Williamson parameter. An augmentation in the magnetic parameter leads to a reduction in velocity, while heightened Biot number and radiation parameter values improve the temperature distribution.
- Lower RMSE values, ranging from ${10}^{-5}$ to ${10}^{-6}$, together with ${R}^{2}\mathrm{=1}$, signify a strong correlation between the machine learning model and the numerical method.
- DNNs have enhanced operational uniformity with a mean deviation of 0.8ms, enabling efficient global sensitivity analysis and immediate heat control.

### Limitations and future research directions

The suggested framework offers a comprehensive computational approach for rapid examination of intricate magneto hydrodynamic nanofluid systems and may facilitate effective parameter evaluation and design-focused research pertinent to thermal-energy applications. However, the current model is constrained by the specified configuration, parameter range, and underlying physical assumptions. In addition, the present computational model comprises simplified physical assumptions about the fluid flow, porous material, and thermophysical characteristics, and additional experimental validation would be required to show its predictive capabilities under actual operating settings. Future endeavors are expected to focus on enhancing the generalization proficiency of the physics-informed surrogate across wider parameter ranges and intricate geometries, in addition to expanding the framework to encompass multi physics scenarios that include supplementary transport mechanisms, transient phenomena, and more authentic porous structures. The exploration of uncertainty aware learning and adaptive sampling techniques can be conducted to enhance the model dependability and resilience in uncertain operational environments.

## Data Availability

The datasets used and/or analyzed during the current study are available from the corresponding author on reasonable request.

## Nomenclature

ML: Machine Learning
ANN: Artificial Neural Networks
DNN: Deep Neural Networks
PINNs: Physics-informed Neural Networks
RMSE: Root Mean Squared Error
PDEs: Partial Differential Equations
tnf: Ternay/Tri-hybrid nanofluid
GSA: Global Sensitivity Assessment
θ: Non-dimensional temperature
ϕ: Concentration profile
${N}_{u}$: The Nusselt number
$${C}_{f}$$: skin friction
ν: kinematic viscosity, $${m}^{2}{s}^{-1}$$
$${C}_{p}$$: specific heat, $$JK{g}^{-1}{K}^{-1}$$
σ: electrical conductivity of fluid, $K{g}^{-1}{m}^{-3}{A}^{2}{s}^{3}$
$${P}_{r}$$: Prandtl number
μ: dynamic viscosity of fluid, $Kg{m}^{-1}{s}^{-1}$
${S}_{c}$: The Schmidt number,
MWCNTs: Multi-walled carbon nanotubes
EG: Ethylene Glycol
W: Water
${\alpha }_{W}$: Williamson fluid parameter
CFD: Computational Fluid Dynamics
AI: Artificial Intelligence
MHD: Magnetohydrodynamics
KBM: Keller-box Method
MAE: Mean Absolute Error
ODEs: Ordinary Differential Equations
hnf: Hybrid nanofluid
$${f}^{\mathrm{''}}$$: Dimensionless Velocity
M: Magnetic parameter
$${B}_{i}$$: Biot Number
(U,V): Velocity components, $$m{s}^{-1}$$
ρ: Density, $$Kg{m}^{-3}$$
${K}_{p}$: The porosity parameter
${F}_{r}$: The Forchheimer number
$${E}_{a}$$: Activation energy
${R}_{d}$: Thermal radiation parameter
η: dimensionless variable
$${\gamma }_{c}$$: Curvature parameter
$${P}_{i}$$: Constants where $$i\mathrm{=1,...4}.$$
${K}_{r}^{2}$: Reaction rate constant
${S}_{Ti}$: Total effect Sobol index
